# CABGen: A Web Application for the Bioinformatic Analysis of Bacterial Genomes

**DOI:** 10.3389/fmicb.2022.893474

**Published:** 2022-05-27

**Authors:** Felicita Mabel Duré, Melise Chaves Silveira, Cláudio Marcos Rocha-de-Souza, Robson Souza Leão, Ivson Cassiano de Oliveira Santos, Rodolpho Mattos Albano, Elizabeth Andrade Marques, Ana Paula D’Alincourt Carvalho-Assef, Fabricio Alves Barbosa da Silva

**Affiliations:** ^1^Central Public Health Laboratory (LCSP), Ministry of Public Health and Social Welfare MSPyBS, Asunción, Paraguay; ^2^Hospital Infection Research Laboratory (LAPIH), Oswaldo Cruz Institute–Oswaldo Cruz Foundation (FIOCRUZ), Rio de Janeiro, Brazil; ^3^Department of Biochemistry, Roberto de Alcântara Gomes Biology Institute, State University of Rio de Janeiro – UERJ, Rio de Janeiro, Brazil; ^4^Department of Microbiology, Immunology and Parasitology - Medical Sciences College - State University of Rio de Janeiro – UERJ, Rio de Janeiro, Brazil; ^5^Scientific Computing Program–Oswaldo Cruz Foundation (FIOCRUZ), Rio de Janeiro, Brazil

**Keywords:** bacteria, web application, pipeline, bioinformatics, genome, antimicrobial resistance

## Abstract

Due to recent developments in NGS technologies, genome sequencing is generating large volumes of new data containing a wealth of biological information. Understanding sequenced genomes in a biologically meaningful way and delineating their functional and metabolic landscapes is a first-level challenge. Considering the global antimicrobial resistance (AMR) problem, investments to expand surveillance and improve existing genome analysis technologies are pressing. In addition, the speed at which new genomic data is generated surpasses our capacity to analyze it with available bioinformatics methods, thus creating a need to develop new, user-friendly and comprehensive analytical tools. To this end, we propose a new web application, CABGen,^1^ developed with open-source software. CABGen allows storing, organizing, analyzing, and interpreting bioinformatics data in a friendly, scalable, easy-to-use environment and can process data from bacterial isolates of different species and origins. CABGen has three modules: Upload Sequences, Analyze Sequences, and Verify Results. Functionalities include coverage estimation, species identification, *de novo* genome assembly, and assembly quality, genome annotation, MLST mapping, searches for genes related to AMR, virulence, and plasmids, and detection of point mutations in specific AMR genes. Visualization tools are also available, greatly facilitating the handling of biological data. The reports include those results that are clinically relevant. To illustrate the use of CABGen, whole-genome shotgun data from 181 bacterial isolates of different species collected in 5 Brazilian regions between 2018 and 2020 were uploaded and submitted to the platform’s modules.

## Introduction

Antimicrobial resistance (AMR) represents a potential threat to human health, being a global concern. A recent study estimated 1.27 million deaths directly attributed to bacterial AMR in 2019 (Murray et al., 2022), and the expectation is 10 million deaths by 2050 (O’Neill, 2016). In addition, the ineffectiveness of antimicrobial treatments increases the number of deaths, hospital length of stay, and healthcare costs (Murray et al., 2022).

Low-income and middle-income countries (LMICs) bear the greatest burden of AMR infections (Abrudan et al., 2021; Murray et al., 2022), with Brazil being a member of this group. One factor that contributes to this scenario is the serious data gaps in many LMICs (Murray et al., 2022). Therefore, controlling AMR in LMICs goes through investing in expanding AMR surveillance and improved technologies, such as whole-genome sequencing (WGS). These efforts should be a part of a global strategy, which can bring universal benefits considering the globalized mobility of the population and livestock (Vegyari et al., 2020).

AMR molecular surveillance using WGS achieves superior reproducibility and resolution compared with other types of molecular-based surveillance. This technology allows, for example, the determination of genes responsible for resistance and virulence, besides understanding pathogen evolution and transmission routes. The information obtained can improve the management of disease outbreaks and epidemics. Furthermore, improved surveillance, with high-quality and standardized data, can reduce unnecessary use of antimicrobials and allow the use of narrow-spectrum drugs, thus reducing selective pressure for resistance (Vegyari et al., 2020).

Challenges, such as the cost of next-generation sequencing (NGS) instruments and reagents, limited knowledge in bioinformatics data analysis, and lack of technical expertise, may impair the implementation of the robust WGS technology (Kumburu et al., 2019). For LMICs, additional challenges are the maintenance and infrastructure costs of a high-performance microbiology laboratory and computational resources, standardization of laboratory information management systems, and the requirement for strict standards at the laboratory level (Vegyari et al., 2020). Despite that, supporting country-specific surveillance systems is an essential global policy recommendation (Adeniji, 2017). Furthermore, each geographic region has its pattern of AMR, and differences can be noticed about the most relevant pathogens and pathogen–drug combinations (Murray et al., 2022).

Understanding the biological complexity of a genome is a first-level challenge, due to the large volume of information generated. With the development and application of NGS technologies, enormous amounts of new data have been and will be increasingly generated. Therefore, it is essential to build platforms that allow organizing, processing, and exploring the data in order to get the most out of the information and improve the understanding of bacterial genomes.

This study effort is a step to overcome the barriers to implementing WGS surveillance for AMR in LMICs. We present a Web Application, with Responsive Web Design technology, that is easy to use, secure, flexible, scalable, and developed with open-source software, that contains a pipeline that is executed sequentially and automatically, called Clinical Applied Bacterial Genomics Analysis System (CABGen).^2^ This application performs WGS analyses from bacterial strains, integrating the use of open-access tools for genomic analysis and data visualization, generating a database available for users.

Although a few pipelines and websites have been developed for bacterial genome analyses (Quijada et al., 2019; Vallenet et al., 2019; Petit and Read, 2020; Sserwadda and Mboowa, 2021), we bring some additional advantages, focusing on clinically applied aspects, easy execution, and functional visualization. The analyses include reads quality; coverage estimation; species identification; *de novo* genome assembly; assembly quality; genome annotation; MLST assignment; search for genes related to AMR, virulence, and plasmids and point mutations detection in specific AMR genes. In addition, CABGen allows users, through authenticated connections, to upload their own paired-end reads from Illumina platforms or FASTA contigs files, to perform genomic analyses, and/or to consult available data already analyzed.

## Materials and Methods

### Collection of Bacterial Isolates

The Laboratório de Pesquisa Em Infecção Hospitalar (LAPIH-FIOCRUZ) takes part, in a National Bacterial Resistance Surveillance Network Headed by The General Coordination of Public Health Laboratories (CGLAB—Brazilian Health Ministry) and receives multidrug-resistant bacteria from different brazilian central public health laboratories that are submitted for molecular analyses. Representative carbapenemase-producing and/or polymyxin-resistant strains, collected between 2018 and 2020 from different states were analyzed here.

### Next-Generation Sequencing

DNA was extracted from bacterial isolates using the QIAamp DNA Mini kit (Qiagen, Germany) and quantified with a QuantiFluor^®^ ONE ds DNA system (Promega, Inc., United States). Sequencing libraries were constructed by transposon tagmentation with the Nextera XT DNA Sample Prep kit (Illumina, United States). Library quantification was performed by fluorometry using the QuantiFluor^®^ ONE ds DNA system and, after proper dilution, the libraries were paired-end sequenced (2 × 250 cycles) using the MiSeq Reagent Kit v2 (500 cycles; Illumina) on a MiSeq instrument. At the end of the run, fastq reads were uploaded to the CABGen pipeline.

### Bioinformatics Pipeline

**Figure 1:**
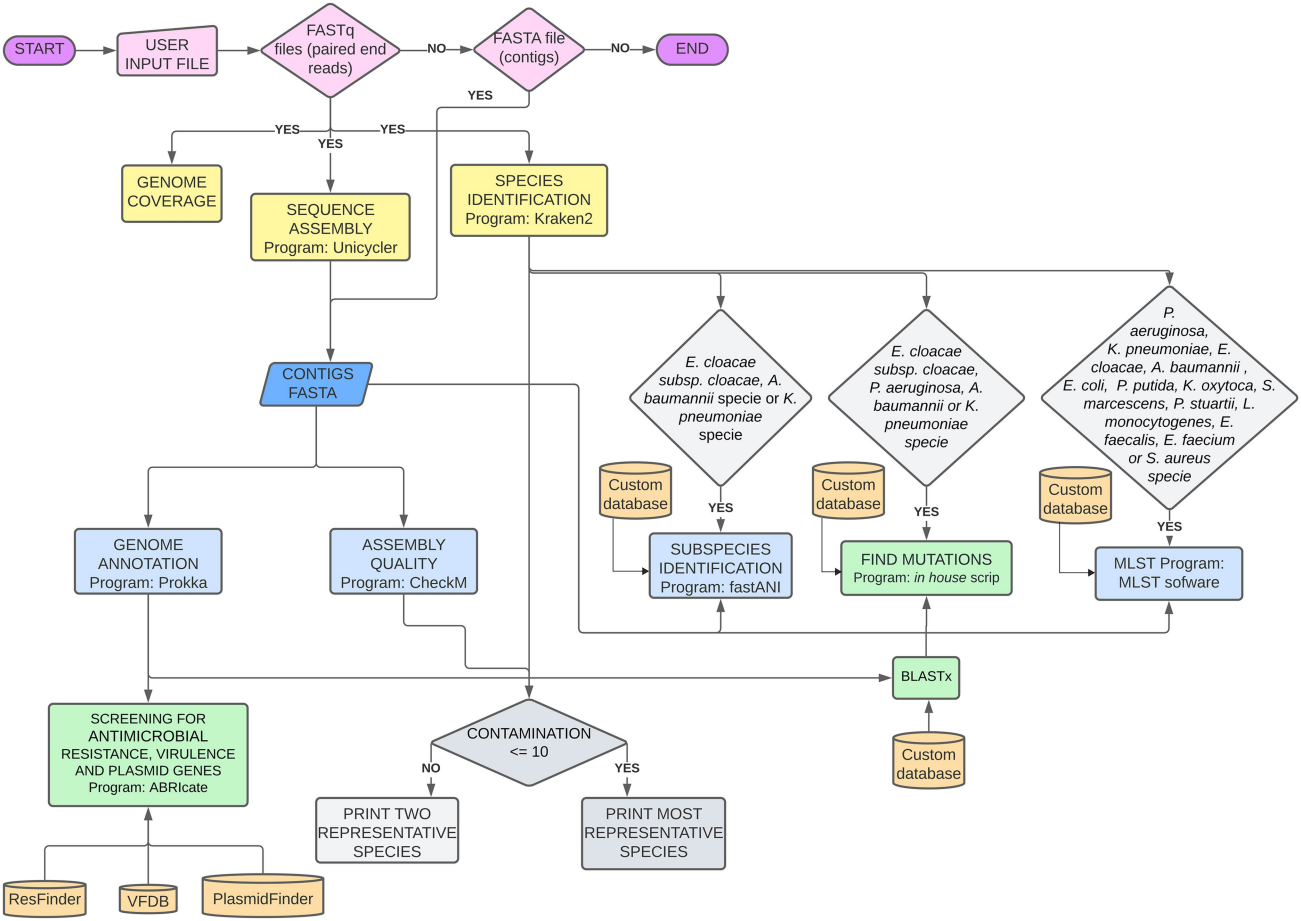
Bioinformatic Pipeline used for genomic sequence analysis. Pink: mandatory files uploaded by the user; Yellow: analyses performed on fastq read files; Blue: analyses performed on fasta contigs files; Green: analyses performed on Prokka outputs; Gray: decisions dependent on two different outputs.

CABGen is based on a bioinformatics pipeline written in the Perl programming language, using a collection of open-source tools and published authoritative databases, such as FASTQC (Andrews et al., 2016), Kraken2 (Wood et al., 2019), FastANI (Jain et al., 2018), Unicycler (Wick et al., 2017), CheckM (Parks et al., 2015), Prokka (Seemann, 2014), ABRIcate,^3^ Resfinder (Zankari et al., 2012), Virulence Factor Database (Liu et al., 2019), and PlasmidFinder (Carattoli et al., 2014), which can be used with bacterial isolates of different species and origins (Figure 1). All programs in the pipeline used default parameters. The command lines used in the pipeline are detailed in the Supplementary Material.

The basic pipeline analyses for any bacterial specie, include reads quality; coverage estimation; species identification; *de novo* genome assembly; assembly quality; genome annotation; and search for genes related to AMR, virulence, and plasmids. For the well-known nosocomial bacterial complex *Acinetobacter baumannii-calcoaceticus*, *Enterobacter cloacae,* and *Klebsiella pneumoniae,* subspecies verification is made by FastANI. MLST assignments was done if the isolate is assigned to one of the following species: *Pseudomonas aeruginosa, K. pneumoniae, E. cloacae, A. baumannii, Escherichia coli, Pseudomonas putida, Klebsiella oxytoca, Serratia marcescens, Providencia stuartii, Listeria monocytogenes, Enterococcus faecalis, Enterococcus faecium, or Staphylococcus aureus.* Point mutations in specific AMR-related genes are detected for *K. pneumoniae, P. aeruginosa*, *A. baumannii*, and *E. cloacae* subsp. *cloacae*.

The user uploads sequence read files in the fastq.gz format and the pipeline executes three steps: (i) reads are *de novo* assembled into a draft genome using Unicycler v0.4.9, which is based on SPAdes (Bankevich et al., 2012) by constructing a De Bruijn graph assembly from k-mers, and Pilon to polish the assembly using short-read alignments (Walker et al., 2014); (ii) species identification, using Kraken v2.0.8-beta (Wood et al., 2019) and FastANI v1.31 (Jain et al., 2018); and (iii) estimation of genome coverage, calculated using the number of paired-end reads multiplied by 250 and divided by genome size. For Kraken, the MiniKraken v2 database was used. After this step, FastANI is also used to confirm species identification for the well-known nosocomial bacterial complexes: *A. baumannii-calcoaceticus*, *E. cloacae,* and *K, pneumoniae*. The strains used to make the FastANI reference databases were: *A. baumannii-calcoaceticus* complex*: A. baumannii* ATCC 19606 (KL810966.1), *A. nosocomialis* NIPH 2119 (KB849239.1), *A. pittii* PHEA-2 (CP002177), *A. calcoaceticus* 2117 (NZ_LS999521); *E. cloacae* complex*: E. asburiae* strain ATCC 35953 (NZ_CP011863.1), *E. bugandensis* isolate EB-247 (LT992502.1), *E. cancerogenus* ATCC 35316 (ABWM02000000.1), *E. chengduensis* strain GN02587 (LEDN01000000.1), *E. cloacae* subsp. *cloacae* ATCC 13047 (NC_014121.1), *E. cloacae subsp. dissolvens* ATCC 23373 (WJWQ01000000.1), *E. hormaechei* subsp. *hormaechei* ATCC 49162 (AFHR01000000.1), *E. hormaechei* subsp*. hoffmannii* DSM 14563 (NZ_CP017186.1), *E. hormaechei* subsp. *oharae* DSM 16687 (NZ_CP017180.1), *E. hormaechei* subsp. *steigerwaltii* DSM 16691 (NZ_CP017179.1), *E. hormaechei* subsp. *xiangfangensis* LMG27195 (CP017183.1), *E. kobei* strain DSM 13645 (NZ_CP017181.1), *E. ludwigii* strain EN-119 (NZ_CP017279.1), *E. mori* LMG 25706 (AEXB01000000.1), *E. roggenkampii* strain DSM 16690 (NZ_CP017184.1), *E. soli* ATCC BAA-2102 (LXES01000000.1); *K. pneumoniae* complex: *K. africana* SB5857 (CAAHGQ010000000.1), *K. pneumoniae* subsp. *pneumoniae* HS11286 (NC_016845.1), *K. quasipneumoniae* subsp. *quasipneumoniae* 01A030T (NZ_CP084876.1), *K. quasipneumoniae* subsp*. similipneumoniae* 07A044T (NZ_CP084787.1), *K. quasivariicola* strain KPN1705 (NZ_CP022823.1), and *K. variicola* SB5531 (CAAHGN010000000.1). The choice of these strains was guided by several articles which considered the strains as taxonomy references (Rodrigues et al., 2019; Wyres et al., 2020; Godmer et al., 2021; Qin et al., 2021; Wu et al., 2021). After assembly, contigs below 500 bp long are discarded and the quality is assessed by CheckM (Parks et al., 2015). The genome size, the number of contigs, the integrity (completeness), and the contamination data produced by CheckM are made available to users in the analysis result tab, which can be accessed through an icon within the system. If contamination is greater than 10%, the two most representative species are shown, with their respective attributed number of reads. Draft genomes are annotated using Prokka (Seemann, 2014), which generates several text-format files *per* genome annotated. The “fna” file generated with Prokka is used for resistance, virulence, and Inc. annotation using ABRIcate^4^ against ResFinder (Zankari et al., 2012), Virulence Factors Database (VFDB; Liu et al., 2019), and PlasmidFinder databases (Carattoli et al., 2014), respectively. The cutoff for BLASTn using ABRIcate is 90% identity and 90% reference sequence coverage. The antimicrobial resistance genes found are classified according to the respective antimicrobial class they can confer resistance to. These results are available to users in the analysis result tab. Since virulence results can be too long and more challenging to interpret, it has an exclusive tab in the individual report. MLST is performed with the mlst software^5^ and the PubMLST database (Jolley et al., 2018), and is also shown in the results tab.

Point mutations are detected by BLASTx alignment of proteins annotated by Prokka against reference sequences. The proteins evaluated for *K. pneumoniae* were from MGH 78578 (CP000647.1) and included protein sequences for PmrA, PmrB, PhoP, PhoQ, MgrB, GyrA, GyrB, ParC, AcrR, and RamR. For *P. aeruginosa*, the strain PAO1 (NC_002516.2) was used as reference and protein sequences analyzed were PmrA, PmrB, PhoQ, ParS, ParR, CrpS, ColR, OprD, AmpC, AmpR, GyrA, GyrB, ParC, and ParE, besides functional MexT from PAO1 Geneva (CAA07694.1). For *A. baumannii* genomes, the proteins PmrA, PmrB, LpxA, LpxC, LpxD, GyrA, GyrB, ParC, AdeM, AdeR, AdeL, AdeS, CarO, and OmpA from ATCC 19606 (CP046654.1) are searched. Lastly, the reference strain *E. cloacae subsp. cloacae* ATCC 13047 is used to analyze protein sequences from PmrA, PmrB, PhoP, PhoQ, MrgB, GyrA, and ParC in *E. cloacae subsp. cloacae*. The output results for proteins related to polymyxin resistance show the amino acid substitution or a truncation status (if the isolated sequence has less than 90% of the reference protein length).

### CABGen Implementation

**Figure 2:**
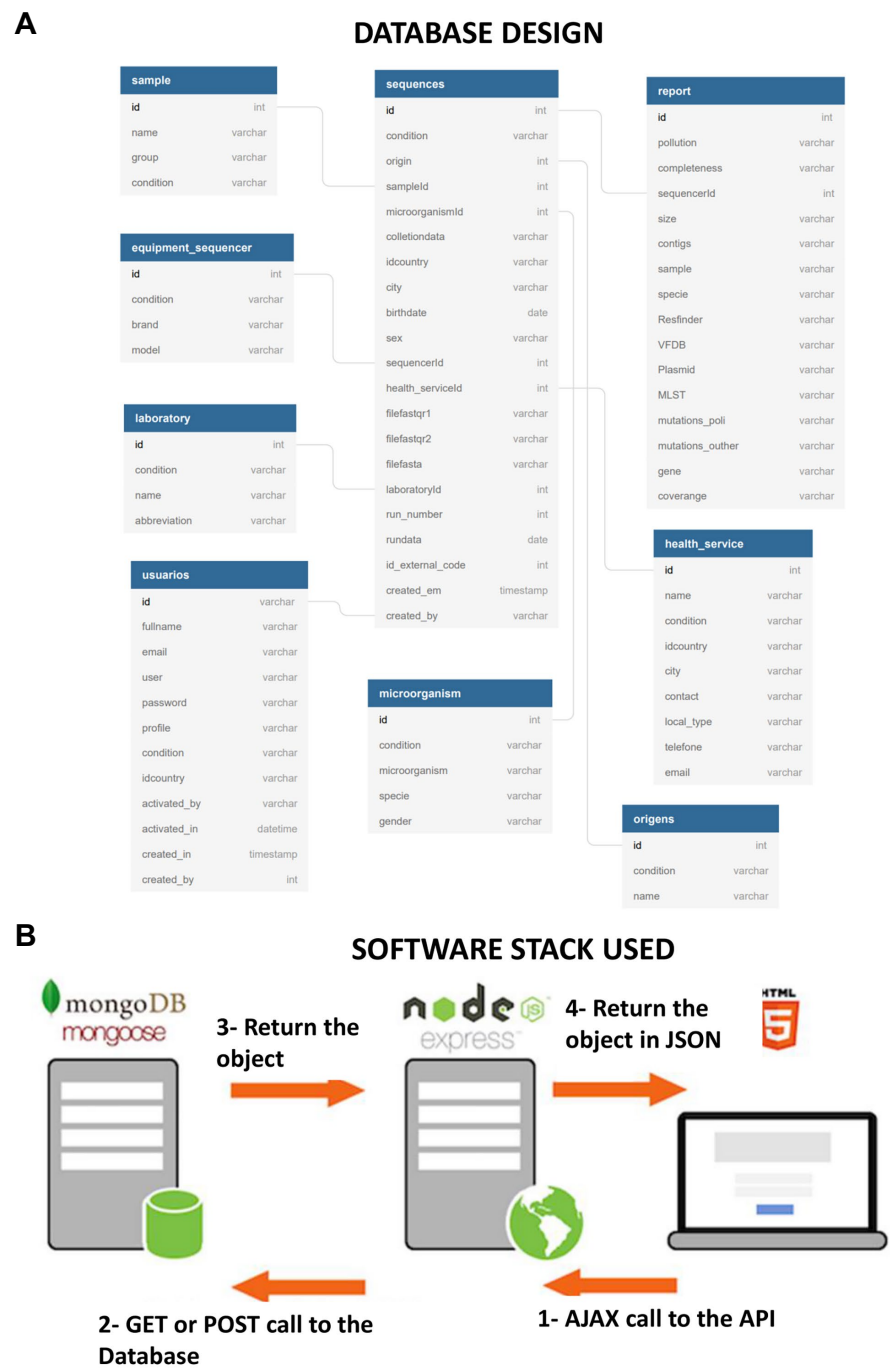
**(A)** NoSql Database Design—MongoDB. **(B)** Web Application Architecture: From the frontend, with html5 we make AJAX calls to our API on the Node server. This consults the database (Mongo) depending on the call made. The DB returns the object as a response to Node and Node serves it as JSON to Html5 that displays it on the frontend without the need to reload the page, thus creating a *Single Page Application*.

The Web application was developed using open-source tools, such as MongoDB (version v4.4.10), a document-oriented, NoSQL database system designed to facilitate application development and scaling^6^; Node.js v10.24.1, that is a cross-platform runtime environment for the server layer based on the JavaScript programming language, asynchronous, with data I/O in an event-driven architecture, designed to create scalable applications, allowing to establish and manage multiple connections at the same time^7^; Express, a back-end web application framework for Node.js designed to build web applications and APIs^8^; and AJAX, short for Asynchronous JavaScript and XML, a web development technique for creating asynchronous web applications, processing any request to the server in the background, interacting with the server without the need to reload the web page, and improving interactivity, speed and usability in applications (Turc, 2019; Figure 2).

CABGen v1.0^9^ is designed to facilitate bioinformatic analysis, with its friendly and easy-to-use environment. It is implemented as a web application, so the user only needs a web browser to access the system. The system interface is intuitive and it is designed with responsive technology that can be used on any device, as well as being available in three languages: English, Spanish and Portuguese. It can be used by users who request access through registration, which sends an email to the application administrators so that they can designate the corresponding profiles: (1) Consultation of Available Data. (2) Consultation and genomic analysis. The return of the user’s authorization is carried out by the same means (Supplementary Material). All programs used are free and installed on a server at PROCC (Fiocruz’s Scientific Computing Program). In this way, the execution of the analysis does not depend on access to other servers.

#### Server Infrastructure

Oswaldo Cruz Foundation in Rio de Janeiro, Brazil, hosts the central CABGen server. The main server technical specifications are: I9 Processor, Hyper-Threading technology with eight cores, 64GB RAM, and 10 TB internal storage disk. Backup copies are made automatically in mirrored internal and external disks. The bioinformatic analysis process is deployed as a scheduled task to be carried out one by one according to the work queue. The system notifies the user of the start and end of the analysis *via* email.

The initial screen of CABGen pipeline has three modules or tasks that will be enabled according to the profile of each user, such as (1) Upload Sequences, (2) Analyze the sequences, and (3) Check the results. In tasks 1 and 2, only the sequences uploaded by the user will be available. All tasks have help legends for users to guide them in their use, in addition to a Support module, where you can access the User’s Manual for the Use of the System and Frequently Asked Questions (Supplementary Material).

Uploading sequences requires a mandatory file: sequence reads in fastq.gz formats or contigs in fasta format. The user must upload one of these formats. Raw paired-end sequences should be obtained from the Illumina platforms. CABGen stores and integrates genomic data and metadata associated with a read. The metadata associated with the genomes is important since it provides invaluable information, such as the type of sample, the microorganism, the geographical location of the health service, the equipment with which it was sequenced, the demographics of the patient, such as the date of birth and sex, and year of isolation (Supplementary Material).CABGen is based on the international ethical principles of the Declaration of Helsinki (Asociación Mundial Médica, 2017) and what was agreed in the 1946 Nuremberg Code (Tribunal Internacional de Núremberg, 1947), which expresses the main purpose of research in human beings, to improve preventive, diagnostic and therapeutic procedures. CABGen cares for the protection of the integrity, intimacy, and confidentiality of the data and guarantees the confidentiality of the information provided by each user. Patient identification data will not be recorded and will be replaced by unique identification codes (sequence number) for purposes of analysis and presentation of results.

The Analyze the sequences module has two tasks: (1) Quality Control and (2) Bioinformatic Analysis. Quality Control (QC) analysis of reads is performed using the free FASTQC software (Andrews et al., 2016), which runs QC checks on raw sequence data from high-throughput sequencing instruments. It provides a modular set of analyzes that users can use to give a quick impression of whether your data has any issues that you should be aware of before performing further analysis. The main features of FastQC are: provide a quick overview to tell you where there may be problems, summary graphs, and tables to quickly assess your data, export results to a permanent HTML-based report, and offline operation to allow automated generation of reports without running the interactive application (Andrews et al., 2016). This report is generated in HTML format, which is available for users by clicking the corresponding icon in the Streams section. To carry out the QC, the reads must be loaded in the System (Supplementary Material).

In the Bioinformatic analysis, the same step is repeated as for the QC analysis, the task must be selected, then the reads to be analyzed (one or more) and confirmed (Supplementary Material). Analyzes include coverage estimation; species identification; *de novo* genome assembly; assembly quality; genome annotation; MLST mapping; search for genes related to AMR, virulence, and plasmids; and detection of point mutations in specific AMR genes. The user is notified *via* email when the results are available.

**Figure 3:**
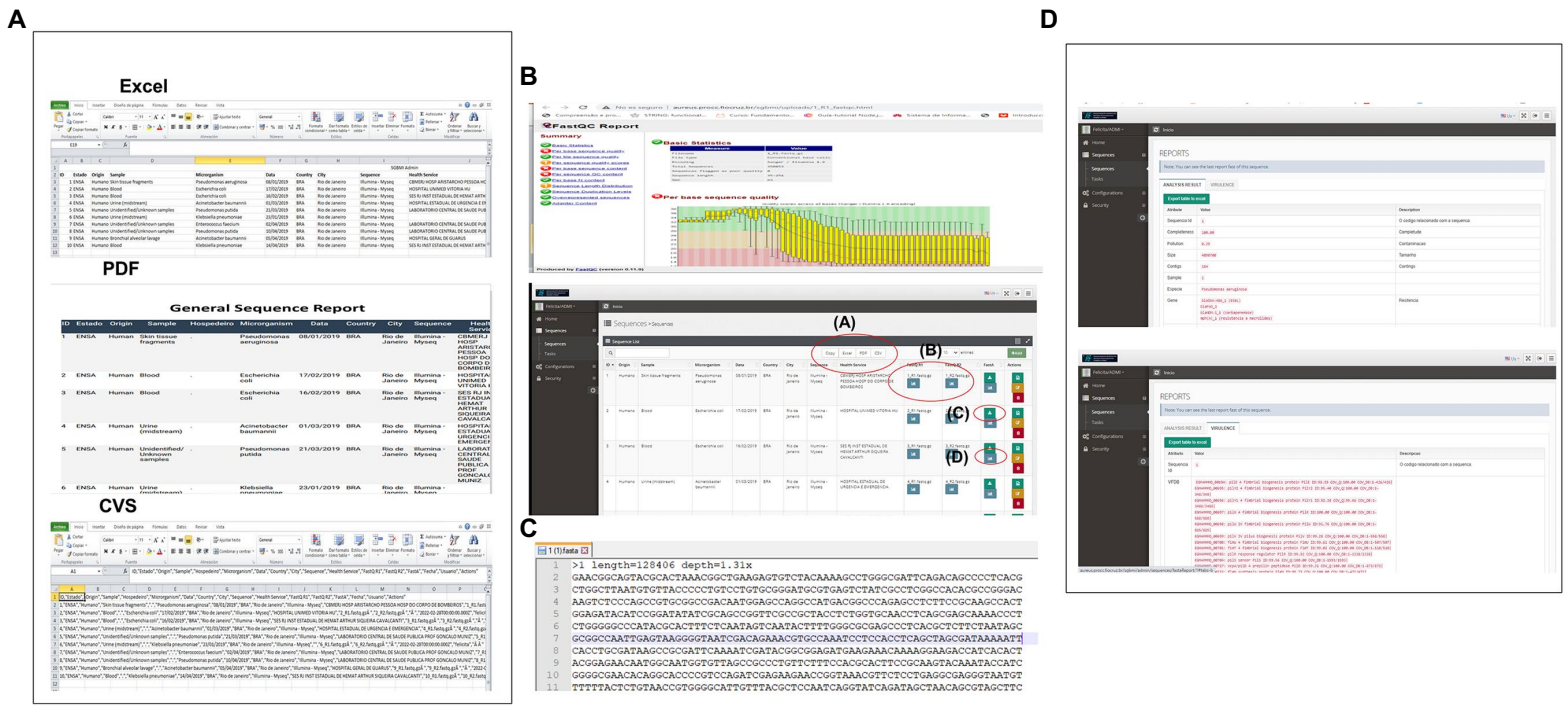
System Output **(A)** In this section, you can download the entire list of user sequences, in Excel, pdf, CVS formats, or just plain text. **(B)** By clicking on this icon you can view the HTML report generated by FASTQC (Sequence Quality Control) in which the user can navigate through the sections offered. **(C)** In this icon, you can access the file in fasta format that is obtained when assembling the reads. **(D)** In this section, you can access the individual report of the sequences with all the results obtained from each of the steps of the Pipeline used.

CABGen supports multiple ways to access data, meeting the needs of both expert and inexperienced computer users. Researchers have the option to select data by browsing or searching the database through the web application user interface. The results can be downloaded. The data available for download are: the raw sequence, the QC analysis, the generated fasta file of the sequence set, the individual sequence results report, as well as the selected data set (Figure 3).

**Figure 4:**
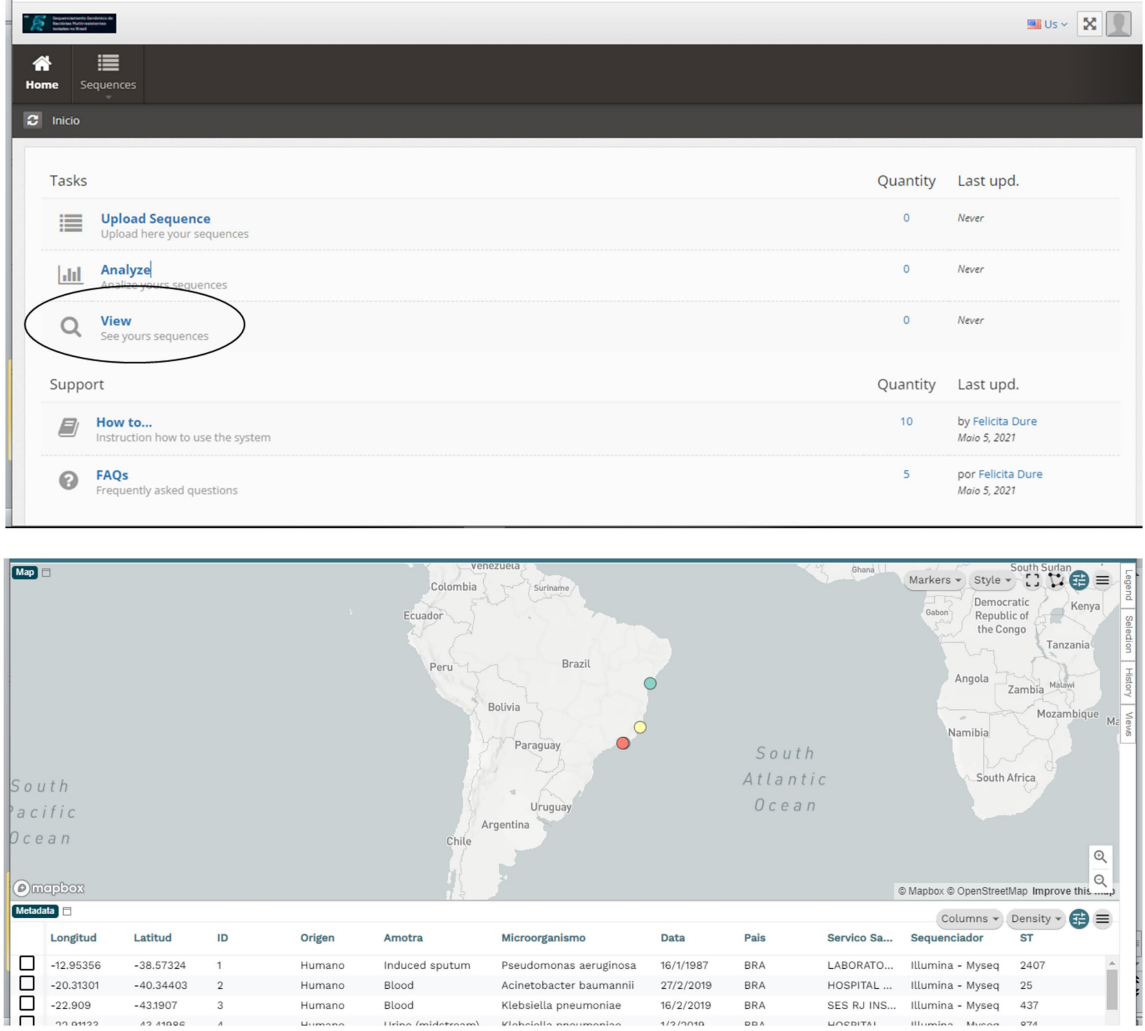
From the Main Menu, in the Queries Task, you can access all the sequenced data. This visualization is done with the Microreact program, which allows visualizing and exploring any combination of grouping (trees), geographic (map), and temporal (timeline) data. Other metadata variables are displayed in a table.

In addition, the system provides a query module where data can be viewed in a georeferenced way using the Microreact sofware (Argimón et al., 2016), which allows users to load, view, and explore any combination of grouping (trees), geographic (map) and temporal (timeline) data. Other metadata variables are displayed in a table. Users can specify colors and/or shapes to display on the map, tree, and/or timeline (Figure 4).

## Results

This section presents the results of the genomic analysis of 181 bacterial isolates from different species, collected from four of five Brazilian regions during the years 2018–2020, and submitted to WGS on an Illumina MiSeq platform.

### Strains Analyzed

Of the 181 genome sequences, 13 were discarded from further analyzes due to contamination as deduced from sequence assembly data. Among the 168 isolates analyzed by the pipeline, 16 species were identified: *K. pneumoniae* (68), *A. baumannii* (34), *P. aeruginosa* (29), *P. putida* (6), *E. hormaechei* [*Enterobacter hormaechei subsp. xiangfangensis* (4), *E. hormaechei subsp. hormaechei* (1) *e E. hormaechei subsp. steigerwaltii* (1)], *E. faecium* (4), *E. coli* (4), *S. marcescens* (4), *E. cloacae subsp. cloacae* (3), *K. oxytoca* (2), *P. stuartii* (2), *Enterobacter nimipressuralis* (1), *Enterobacter bugandensis* (1), *Enterobacter chengduensis* (1), *Enterobacter asburiae* (1), *Pluralibacter gergoviae* (1), and *Enterobacter* (1). Among the most frequent species, an average genome size of 5,718,819 bp was obtained for *K. pneumoniae*, 4,035,922 bp for *A. baumannii*, and 7,030,622 bp for *P. aeruginosa*, thus obtaining the expected genome size range for these species. Regarding data completeness, 99.9% were obtained for *K. pneumoniae* and *A. baumannii*, and 99.5% for *P. aeruginosa*.

**Figure 5:**
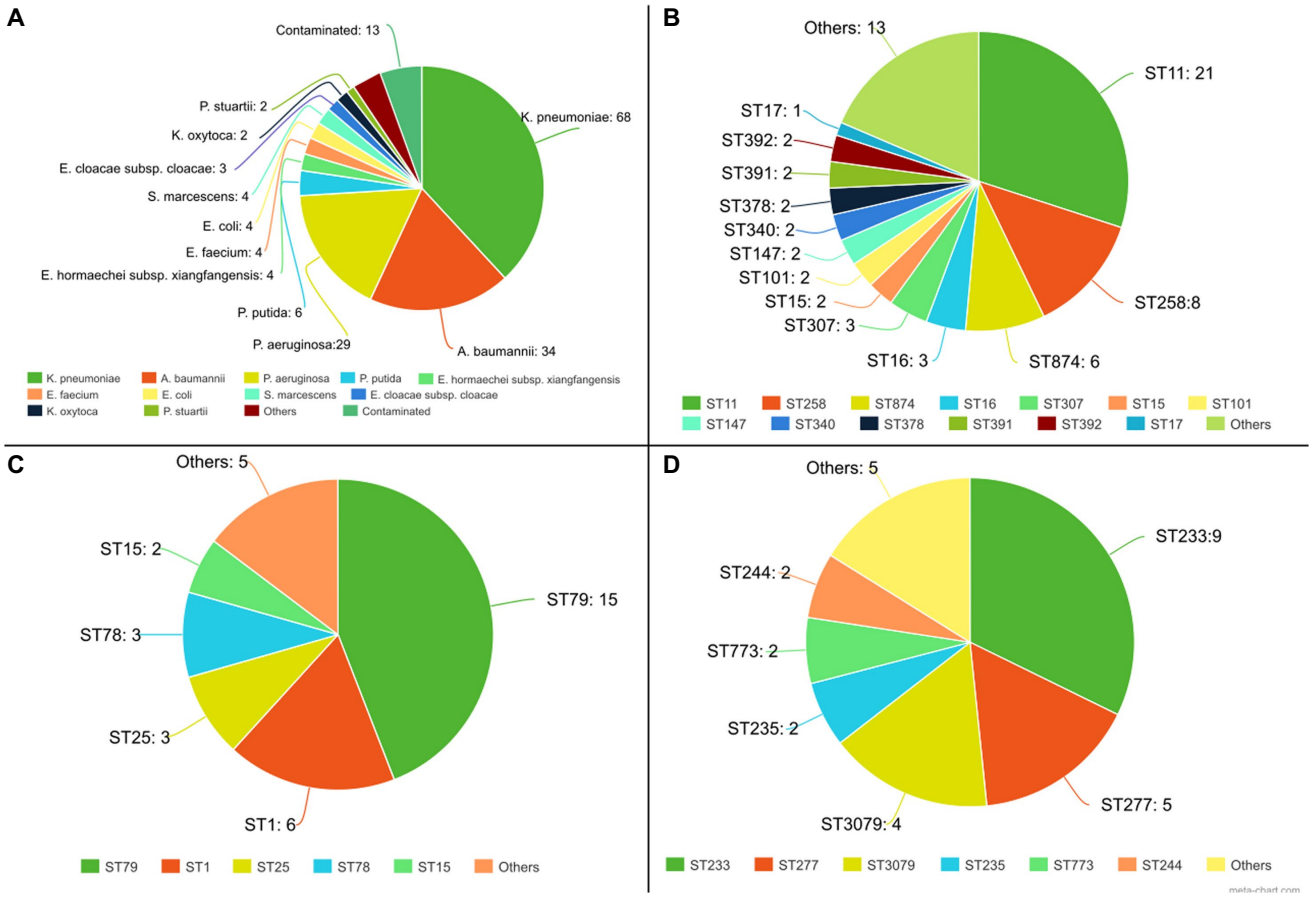
Part of the results generated by the analyses pipeline from 181 sequenced genomes. **(A)** Species identification from 181 isolates; **(B)** STs for *K. pneumoniae* isolates; **(C)** STs for *A. baumannii* isolates; **(D)** STs for *P. aeruginosa* isolates;

**Figure 6:**
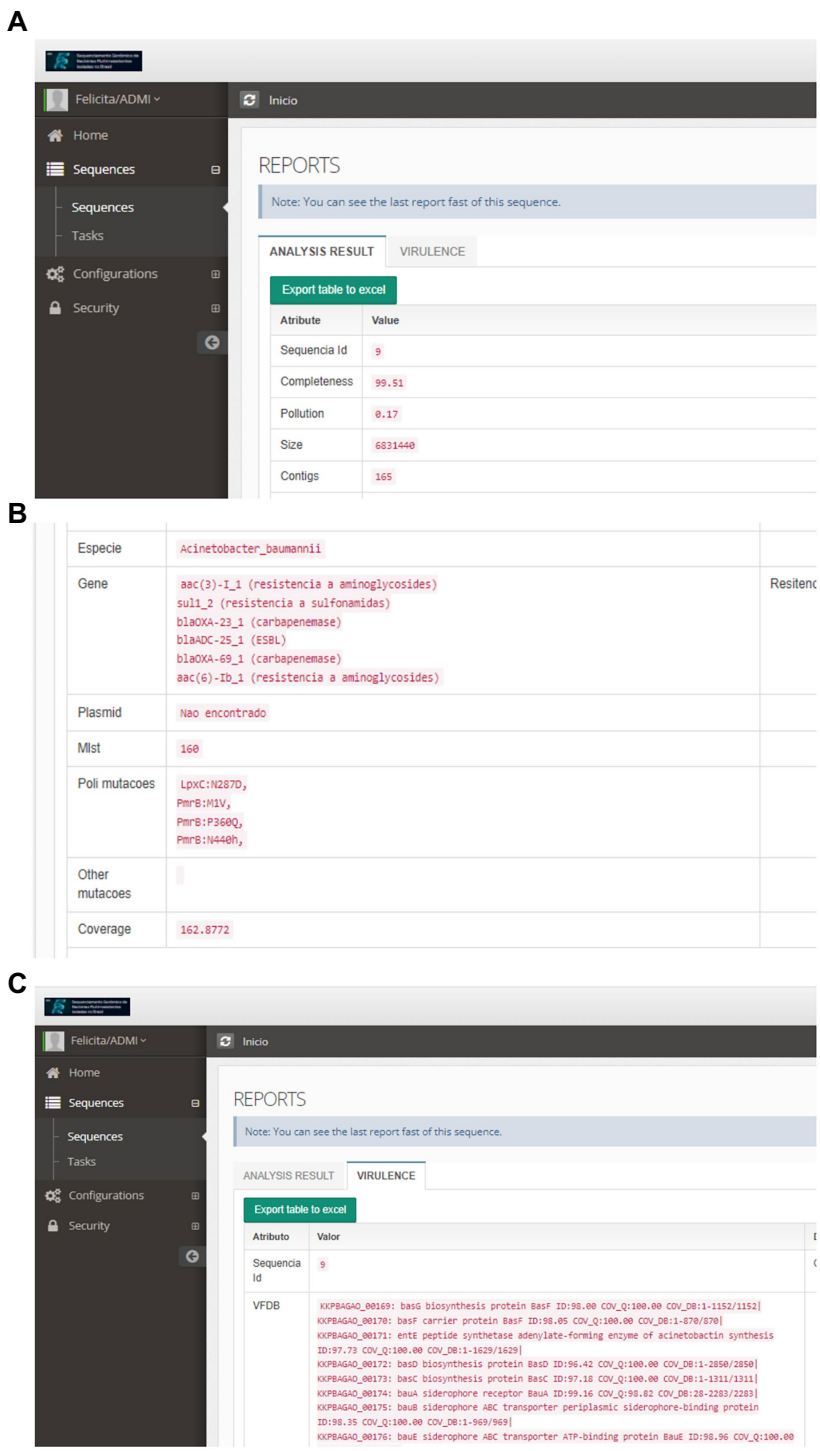
Individual Report of the Result of the Bioinformatic Sequence Analysis: **(A)** Assembly Quality Control. **(B)** Genes Detected, Plasmids, MLST, and Mutations **(C)** Virulence.

For these species, the isolates belonged to different sequence types (STs), and the most frequent clones were ST11 (*n* = 21) and ST258 (*n* = 8), for *K. pneumoniae*, ST79 (*n* = 15) and ST1 (*n* = 6) for *A. baumannii*, and ST233 (*n* = 9), ST277 (*n* = 5) and ST3079 (*n* = 4) for *P. aeruginosa* (Figure 5). Considering the analysis of resistance genes, the result contains the gene name and the class of antimicrobials to which it can confer resistance. For 110 isolates, at least one Inc. group was assigned. Virulence genes’ diversity found for the analyzed isolates was very large. For each isolate, a list of proteins associated with virulence is provided. In this tab, it is possible to verify the protein identifier, its functional description, and identity with the database sequence, in addition to the isolate and database sequence coverage. Mutation results were divided between genes related to polymyxin and genes related to other antimicrobial classes. The dataset generated for mutations allows the user to evaluate the frequency and association of mutations with the resistance profiles, facilitating further studies that phenotypically prove the influence of these genetic alterations on the isolates’ antimicrobial resistance. The data provided by the pipeline could be explored, like a previous manuscript published by the authors, which described 84 Gram-negative bacilli isolated from bloodstream infections in Brazil (Silveira et al., 2021). CABGen generates a report where these results can be viewed, greatly facilitating the handling of biological data, thanks to the use of tools that gather, store, organize, analyze and allow the interpretation of these data. It is essential that the results are communicated in a clear, consistent, and concise manner since the reports can be read by either expert or inexperienced personnel in high-throughput sequencing. To this end, these reports only include those results that are clinically relevant (Figure 6).

## Discussion

All tools and databases used for data analysis, visualization, and website construction are open. This characteristic saves costs and counts on the global community for quality improvement due to constant use and open forums.

Several pipelines have been developed for bacterial genome analyses, especially in recent years (Quijada et al., 2019; Petit and Read, 2020; Xavier et al., 2020; Sserwadda and Mboowa, 2021). Every pipeline has strengths and limitations in the analyses they provide, data visualization, execution, and updating policy. Some pipelines that run on the user’s local machine do not infer quality, like Torme (Quijada et al., 2019). Most of them do sequence quality filtering (Quijada et al., 2019; Petit and Read, 2020; Xavier et al., 2020; Sserwadda and Mboowa, 2021), but we choose not to do this step once the parameters adopted can be very relative and strict filtering may cause loss of essential genes. We think that the user can evaluate FastQC intuitive reports and decide to run, or not, the pipeline. Analyses performed by our pipeline are species identification (not performed by rMAP and Bacpipe; Xavier et al., 2020; Sserwadda and Mboowa, 2021), assembly quality (not performed by rMAP; Sserwadda and Mboowa, 2021), and point mutations detection in specific AMR genes for *K. pneumoniae, P. aeruginosa*, *A. baumannii*, and *E. cloacae* subsp. *cloacae* (not performed by the other aforementioned pipelines; Quijada et al., 2019; Petit and Read, 2020; Xavier et al., 2020; Sserwadda and Mboowa, 2021). Furthermore, regarding updating of the evaluated pipelines, the most recent version varies from 2019 (Bacpipe), 2020 (rMAP), 2021 (Torme), and 2022 (Bactopia).

Most pipelines are built up to run locally, and the output formats are directories and tables (Bacpipe and Bacptopia; Petit and Read, 2020; Xavier et al., 2020) or web-like reports (rMAP and Torme; Quijada et al., 2019; Sserwadda and Mboowa, 2021). Like CABGen, other online implemented pipelines are also available, but they are more complex and can generate lots of data (Wattam et al., 2014; Vallenet et al., 2019).

MicroScope starts with the user submission of assembled genomes and metagenomes. Then it has as priority the annotation of gene functions through genomic, pangenomic, and metabolic comparative analysis. The program uses several tools and databases through the “MicroScope automatic annotation pipeline.” The genomes submitted pass through several analyses’ workflows. Users can also make a systematic and efficient revision of the annotated result (Vallenet et al., 2019). CABGen prioritizes the annotation of acquired AMR genes using the ResFinder database, while Microscope is based on the CARD database, which provides broader data, which may not be directly related to AMR. Besides, MicroScope does not annotate Inc. genes, does not identify the genome sequence type, and only well-known mutations of AMR genes are annotated.

PATRIC, the bacterial bioinformatics resource center, makes complex analyses, providing a wide range of services and tools for genomics, metagenomics, transcriptomics, metabolomics, and protein data. Genome assembly can be made upon different sequencing technologies’ outputs. Furthermore, every genome available at PATRIC is annotated using Rapid Annotations using Subsystems Technology (RAST). Besides, there are thousands of bacterial genomes available in PATRIC (Wattam et al., 2014). PATRIC has several analysis pipelines for bacterial genomes, and the “Comprehensive Genome Analysis Service” is the most straightforward, providing a streamlined analysis that accepts raw reads. However, compared to CABGen, PATRIC’s straightforward analysis pipeline does not include assembly evaluation, annotation focused on AMR, plasmids, virulence, or mutation identification.

Web server-based analysis depends on the server load and requires a fast and consistent internet connection to upload sizable raw data files. Also, the analyses are preformatted and do not allow user customization, and they require the data to be uploaded into their servers (Wattam et al., 2014). However, it has several advantages: no need for local software installation and configuration, an automatic version updating process, and no specific hardware requirements.

Considering the analyses of AMR genes, CABGen has relevant differences from other platforms. First, it is focused on acquired resistance genes, once intrinsic chromosomal resistance genes, like efflux pumps, are predictable according to each bacterial species and can alter species-specific resistance profiles only when regulators pass through specific mutations (Maseda et al., 2010). Exclusively exploiting the resistome of pathogenic bacteria, which is done by rMAP and other pipelines (Sserwadda and Mboowa, 2021), does not focus on genes that alter the antimicrobial prescription. Second, CABGen looks for SNVs in essential genes implicated in AMR, particularly for polymyxin, which is one of the last choice antimicrobials with considerable resistance rates, although the resistance is often not related to acquired genes (Poirel et al., 2017). Third, the prediction of AMR phenotypes does not rely on the presence or absence of resistance genes alone but several other aspects are considered, like mutations in specific genes. Finally, a more significant sample number is crucial (Kumburu et al., 2019) to identify mutation trends. So, this particular data provided by CABGen is very relevant considering clinical applications.

Another critical function of CABGen is the discrimination of bacterial species complex. The pipeline adopted fastANI, which provides ANI (Average Nucleotide Identity) between pairs of genomes. This method has been used to define species for *Enterobacter cloacae* complex (Sutton et al., 2018), *Acinetobacter* spp. (Qin et al., 2021) and *K. pneumoniae* complex (Rodrigues et al., 2019).

The metadata collected and provided by CABGen in the visualization step is very useful. With the avalanche of available and projected genomic data, this type of metainformation is essential to classify and find genomes of interest (Wattam et al., 2014).

## Concluding Remarks

CABGen is an easy-to-use, scalable web application applicable to bacterial samples of different species and origins. This tool dramatically helps users with little knowledge of bioinformatics and programming, since the automation of its pipeline makes it easier to obtain results in a user-friendly format. In future versions, we will incorporate RNAseq analysis. Our final goal is to make GABGen a complete tool for the genomic analysis of bacteria applied to the clinic.

## Availability and Requirements

Project name: GABGen.

Project home page: https://aureus.procc.fiocruz.br/

Operating system: for example, Web-based, Platform independent.

Programming language: Node.js, Perl.

Other requirements: An updated web browser (e.g., Google Chrome, Mozilla Firefox, and Microsoft Edge).

License: Not Applicable.

Any restriction to use by non-academics: Not Applicable.

The user must provide a fastq.gz file (R1 and R2) or contigs in fasta format to analyze it. In order to use the system, you must be registered. The user assignment is sent *via* email.

## Data Availability Statement

The datasets presented in this study can be found in online repositories. The names of the repository/repositories and accession number(s) can be found at: NCBI BioProject—PRJNA677881.

## Author Contributions

FD and MS designed and developed the system. FS and AC-A supervised and approved the development. RA, CR-d-S, RL, IO, and EM performed WGS of the samples. All authors have read and approved the writing of this article.

## Funding

The authors thank CNPq FAPERJ, FIOCRUZ, CAPES, UERJ and UFF. Project: Whole-genome sequencing as a tool to evaluate the propagation of different resistance mechanisms and circulating clones of multiresistant bacteria from different Brazilian states, FAPERJ: E-26/202.554/2019 and Projeto Redes E-26/211554/2019 CNPq/DECIT: 402524/2018–7, for financial support.

## Conflict of Interest

The authors declare that the research was conducted in the absence of any commercial or financial relationships that could be construed as a potential conflict of interest.

## Publisher’s Note

All claims expressed in this article are solely those of the authors and do not necessarily represent those of their affiliated organizations, or those of the publisher, the editors and the reviewers. Any product that may be evaluated in this article, or claim that may be made by its manufacturer, is not guaranteed or endorsed by the publisher.

## Footnotes

^2^ https://aureus.procc.fiocruz.br/

^3^ https://github.com/tseemann/abricate

^4^ https://github.com/tseemann/abricate

^5^ https://github.com/tseemann/mlst

^6^ https://docs.mongodb.com/manual/

^7^ https://nodejs.org/en/

^8^ https://expressjs.com/pt-br/

^9^ https://aureus.procc.fiocruz.br/
