# Supplementary material for: CABGen: A Web Application for the Bioinformatic Analysis of Bacterial Genomes
Source: Front Microbiol. 2022 May 27;13:893474. doi: 10.3389/fmicb.2022.893474 (PMC9196194; doi:10.3389/fmicb.2022.893474)

SYSTEM USER MANUAL

CABGen v1.0

Clinical Applied Bacterial Genomic Analysis System

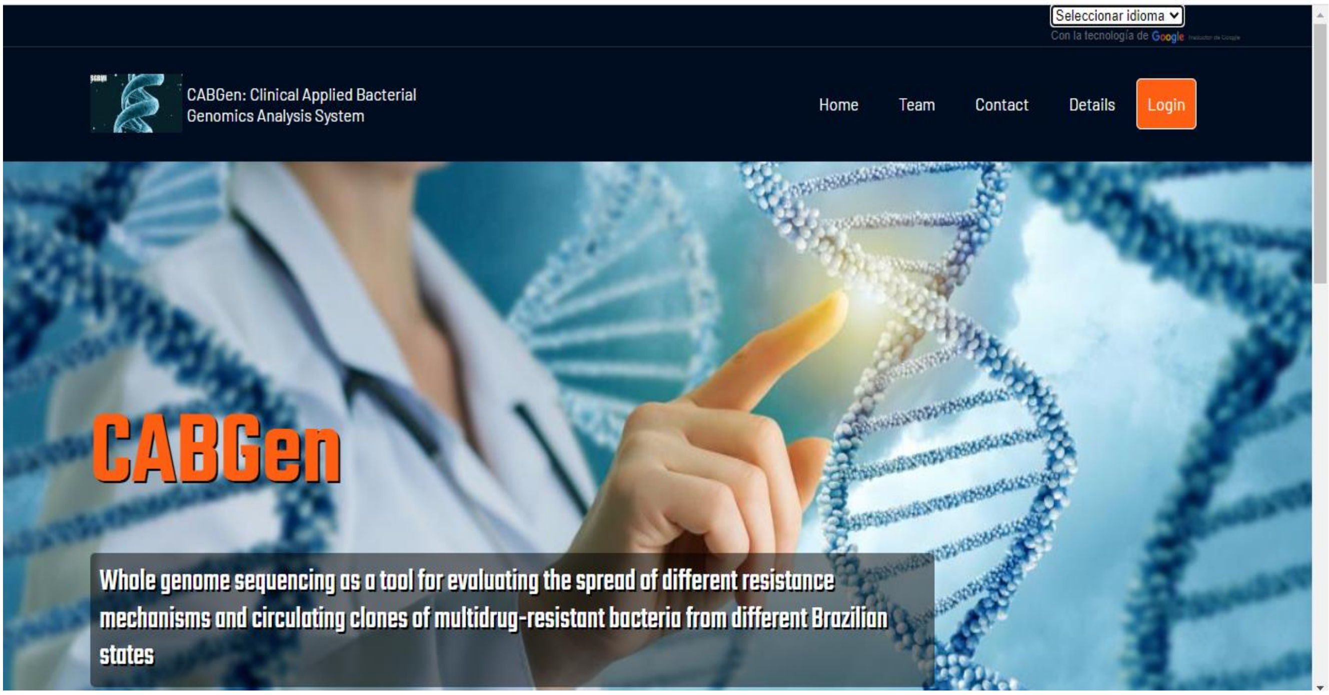

March 2022

PROCC – FIOCRUZ

RIO DE JANEIRO - BRAZIL

CABGen v1.0

SYSTEM USER MANUAL

Content

1. System description 3

2- Start using CABGen. 5

3- Enter the CABGen System. 5

4- Main Menu 6

5- Taks Section: The Tasks Section has 3 processes: 7

5.1 Upload Sequences 7

5.2 Analyze Sequences: 9

a. Quality Control: 9

b. Bioinformatic Analysis: 10

c. Visualization of Results: 13

5.3 Viewss: 17

6. Support 18

1. System description

The web application was developed using open source tools such as: MongoDB (version v4.4.10), a document-oriented NoSQL database system designed to facilitate application development and scaling (https://docs.mongodb.com/manual/); Node.js v10.24.1, which is a cross-platform runtime environment for the server layer based on the JavaScript programming language, asynchronous, with data I/O in an event-based architecture, designed to create scalable applications, allowing to establish and manage multiple connections at the same time (https://nodejs.org/en/); Express, a back-end web application framework for Node.js designed to create web applications and APIs (https://expressjs.com/pt-br/); and AJAX, short for Asynchronous JavaScript and XML, a web development technique for creating asynchronous web applications, processing any requests to the server in the background, interacting with the server without reloading the web page, and improving interactivity, speed, and usability in applications.

CABGen v1.0 (https://aureus.procc.fiocruz.br/) is designed to facilitate bioinformatics analysis, with its friendly and easy-to-use environment. It is implemented as a web application, so the user only needs a web browser to access the system. The system interface is intuitive and designed with responsive technology that can be used on any device, as well as being available in three languages: English, Spanish and Portuguese. It can be used by users who request access through the registry, which sends an email to the administrators of the application so that they can designate the corresponding profiles: 1 - Consultation of Available Data. 2- Consultation and genomic analysis. The return of the user's authorization is carried out by the same means. All programs used are free and installed on a server in PROCC (Fiocruz's Scientific Computing Program). In this way, the execution of this analysis does not depend on access to other servers.

CABGen is based on a bioinformatics pipeline written in the Perl programming language, using a collection of open source tools and published authoritative databases such as FASTQC, Kraken2, FastANI, Unicycler, CheckM, Prokka,

ABRIcate, Resfinder, Virulence Factor Database and PlasmidFinder, which can be used with bacterial isolates from different species and origins.

Analyses include the quality of readings; coverage estimation; species identification; *de novo genome* assembly; assembly quality; genome annotation; MLST assignment; search for genes related to AMR, virulence and detection of plasmids and point mutations in specific ADR genes. In addition, **CABGen** allows users, through authenticated connections, to upload their own paired end readings from Illumina platforms or FASTA contigs files, perform genomic analysis and/or query available data already analyzed.

The initial **CABGen** screen has three modules or tasks that will be enabled according to each user's profile, such as:

1- Load sequences
2- Analyze the sequences
3- Verify the results.

In tasks 1 and 2, only user-uploaded sequences will be available. All tasks have help legends for users to guide them in their use, in addition to a Support module, where you can access the User Manual for the Use of the System and Frequently Asked Questions.

2- Start using CABGen.

To start using the **CABGen** system you must access the following address: **(https://aureus.procc.fiocruz.br/)** In this way, you will see the following screen

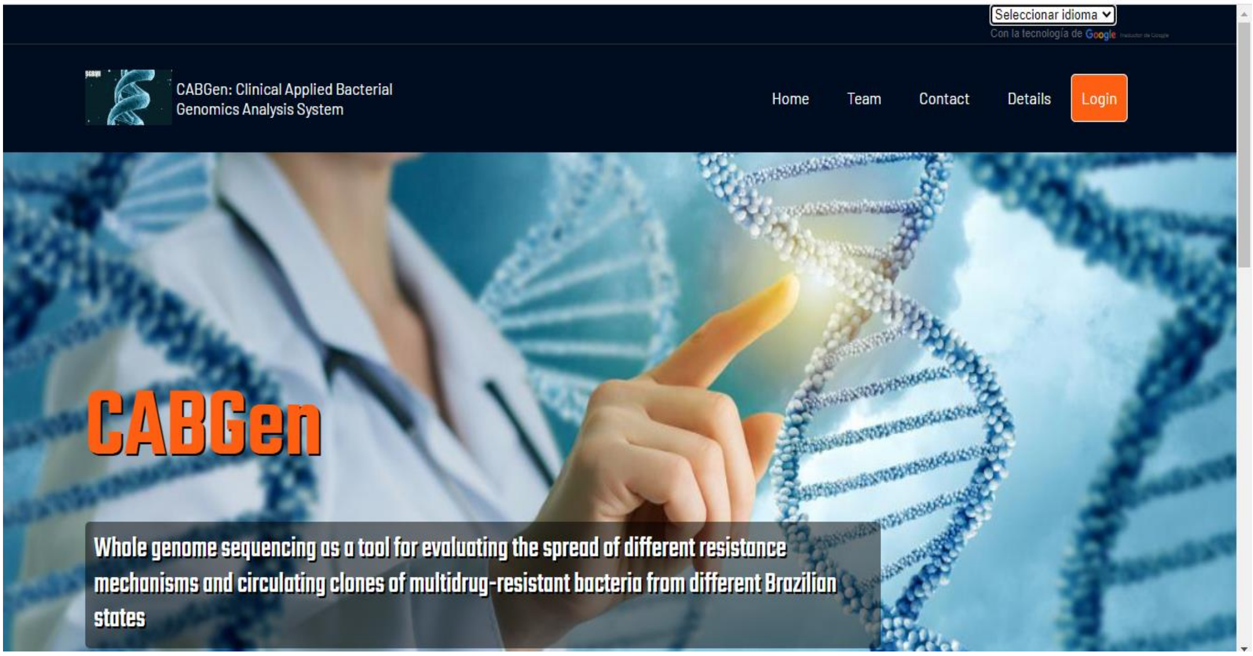

On this screen, you must click on the Login icon to access the **CABGen** System.

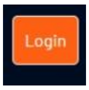

3- Enter the CABGen System.

To enter the System you must have a User and a password, provided by the System Administrator.

To obtain the username and password you must register, clicking on the REGISTER button 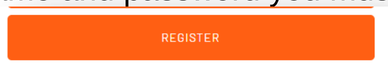

, which will take you to a form so you can enter your data and save them.

This Registration request will be analyzed by the System Administrators and will have a return via email of the confirmation of Creation and user authorization in a period not exceeding 48 hours

.

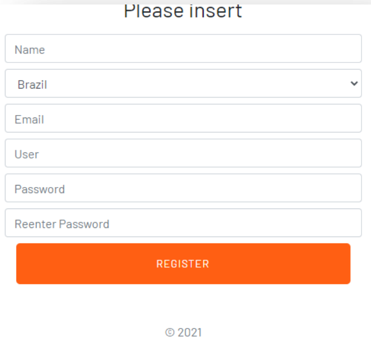

If you already have a username and password you must enter the data in the enabled fields and click on LOGIN.

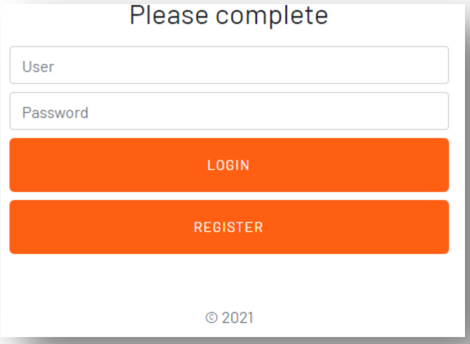

4- Main Menu

The CABGEn Main Menu has the following blocks:

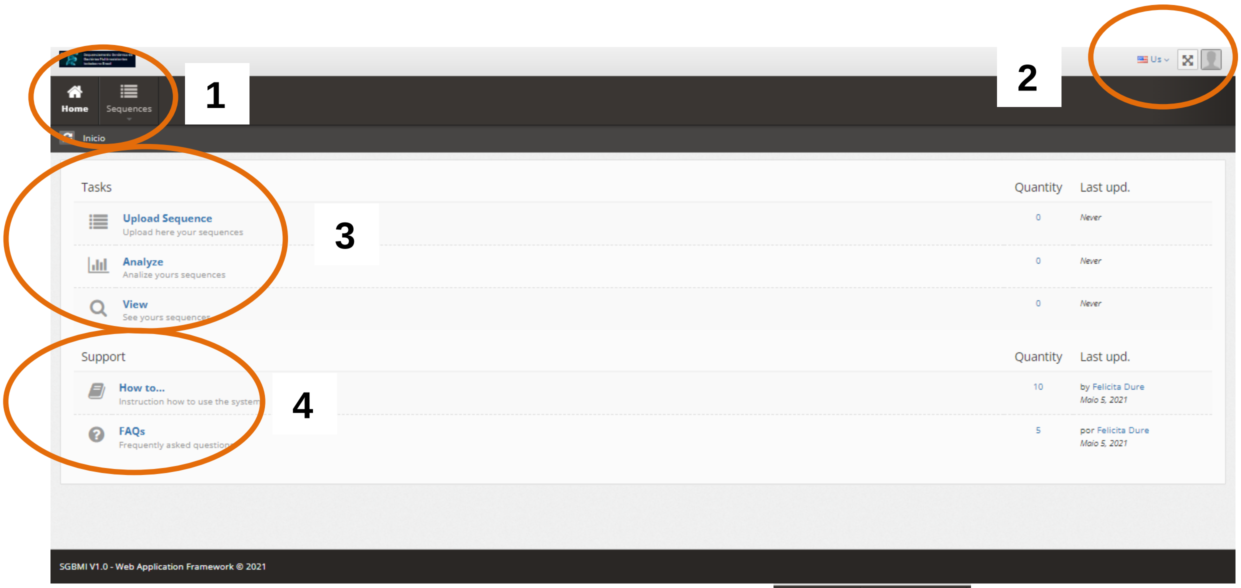

1- Quick Access Icons (Start - Sequences)

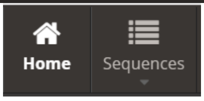

2- Icons for:

a. Change Language

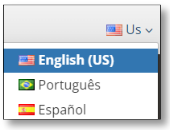

b. Expand the Sreen

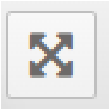

b. Exiting the System

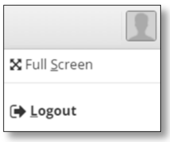

3- Tasks :
   a. Upload Sequences
   b. Analyze
   c. Views

4- Support

a. How to

b. Frequently Asked Questions (FAQs)

5- **Taks Section:** The Tasks Section has 3 processes:

5.1 Upload Sequences: In this task, the files in fastq.gz or fasta format of the sequences are uploaded. The user must first enter the metadata of the sequence to be analyzed, which are:

a. Origin: It refers to whether the sample is of animal, human, environmental, etc. origin.

b. Sample Type: You must select the sample type according to a preloaded list Example: Blood, Urine, Skin Lesion, Cerebrospinal Fluid, etc.

c. Microorganism: You must select the microorganism I suspect from a preloaded list.

d. Country: You must select the country of origin of the sample.

and. City: You must write the city where the sample comes from.

F. Sequencer: You must select the sequencing equipment where the sample sequencing run was performed.

g. Laboratory: You must select the laboratory in charge of performing the sequencing.

h. Health Service. You must select the health service which sent the sample.

i. Date of Birth: It is the date of birth of the patient, this if the origin of the sample is Human.

J. Gender: The patient's gender if applicable.

k. Origin Code: It is the origin code of the sample.

l. Sample Collection Date: The date the sample was drawn or taken.

m. Run Number: It is the run number of the sample sequencing.

n. Sequencing Date: It is the date that the sequencing of the sample was performed.

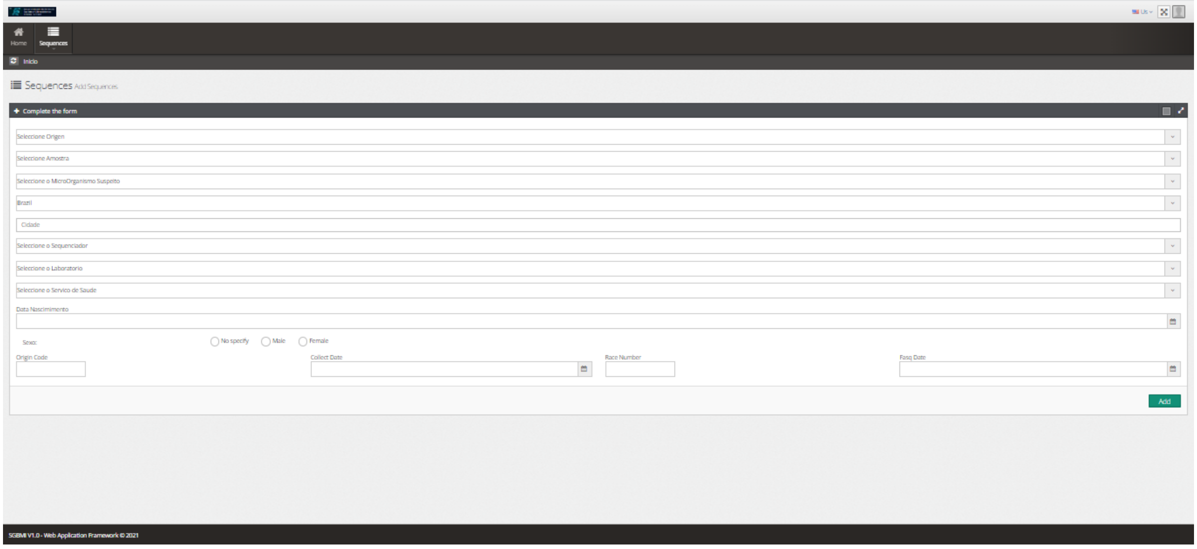

Once all the data has been entered, you must click on the Add button, 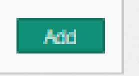

in order to upload the files. The following screen appears:

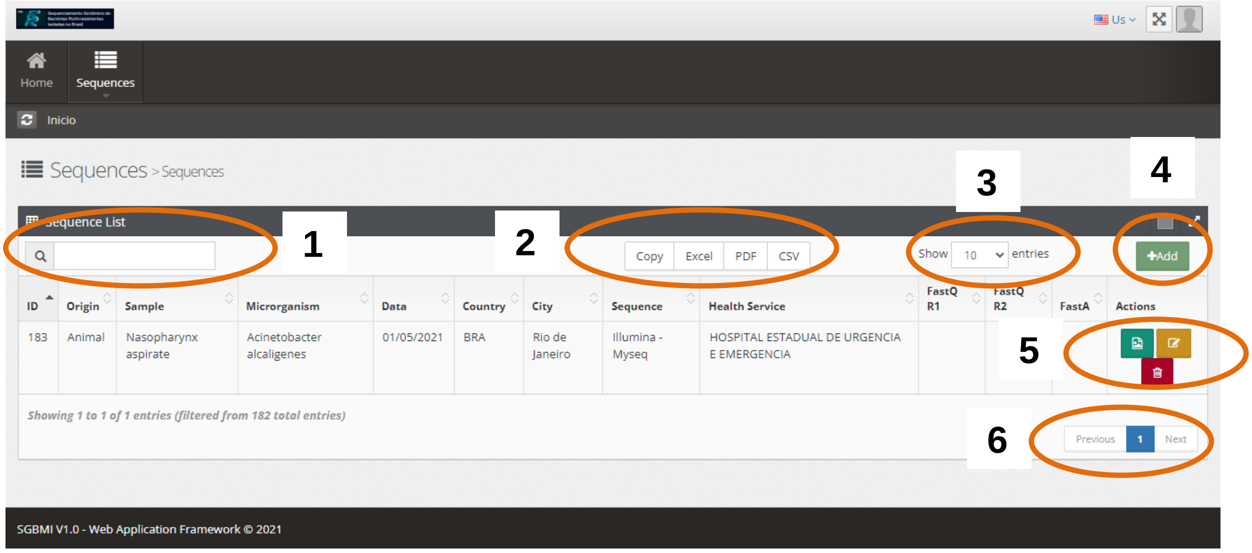

Where there are several icons such as:

1- Search engine: With this icon you can search for sequences.

2- With these icons you can download the list of uploaded sequences in Excel, PDF and CSV formats, how to copy all the data.

3- In this section you can select the number of records to be displayed.

4- Icon to add a new record.

5- This group of icons has several functions:

a. Add files.

b. Edit file.

c. Delete file

6- Sequence scroll bar.

To upload files, the accepted files are:

- Raw readings generated in .fastq.gz format that must be uploaded separately for R1 and R2.
- Or files in fasta format.

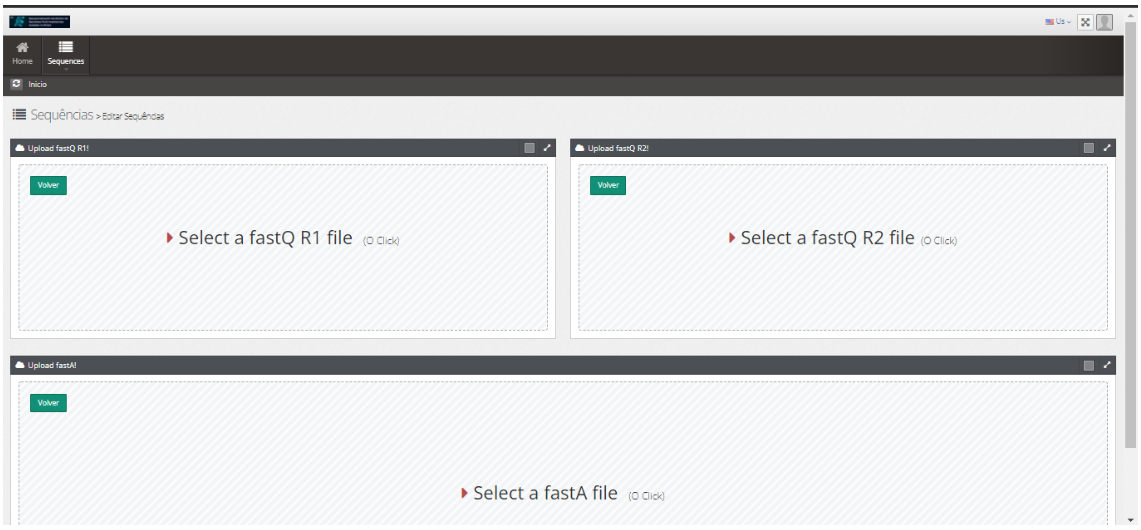

Once the sequences have been uploaded, the name of the already renamed sequences uploaded appears in the FastQ R1 and FastQ R2 column.

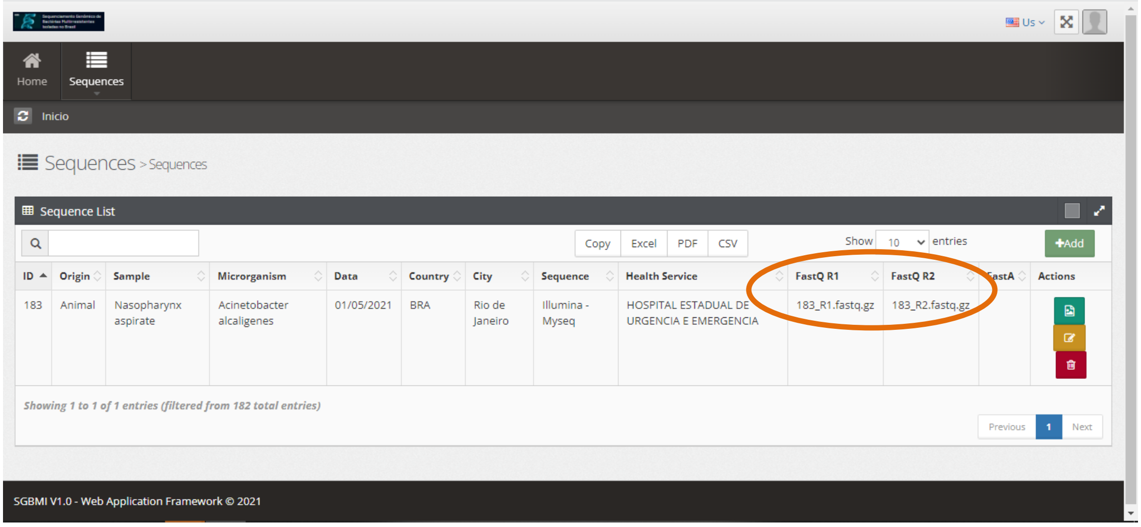

5.2 Analyze Sequences: In this section, two types of analysis are carried out with the sequences uploaded by the user, which are:

a. Quality Control: This analysis consists of performing quality control on the raw sequences using the FASTQC program, selecting the Quality Control task.

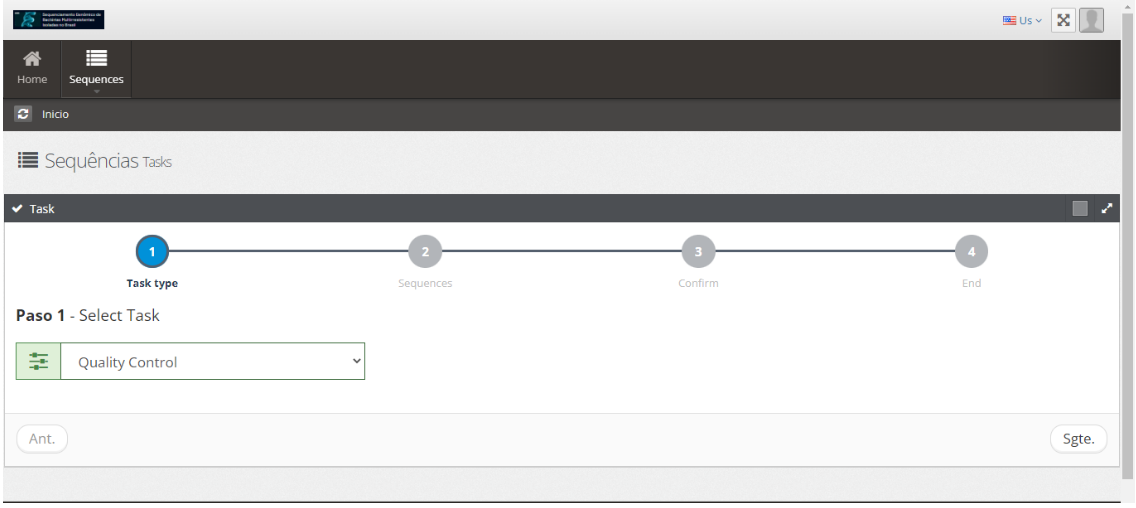

b. **Bioinformatic Analysis:** This analysis consists of the complete analysis of the sequence, which can be performed for one or more sequences; In this task, the following processes are performed:

   a. Coverage estimate
   b. Species identification
   c. Genome assembly (de novo)
   d. Assembly quality
   e. Genome Annotation
   f. MLST Assignment
   g. Search for AMR-related genes, virulence and plasmid detection and point mutations in specific AMR genes.

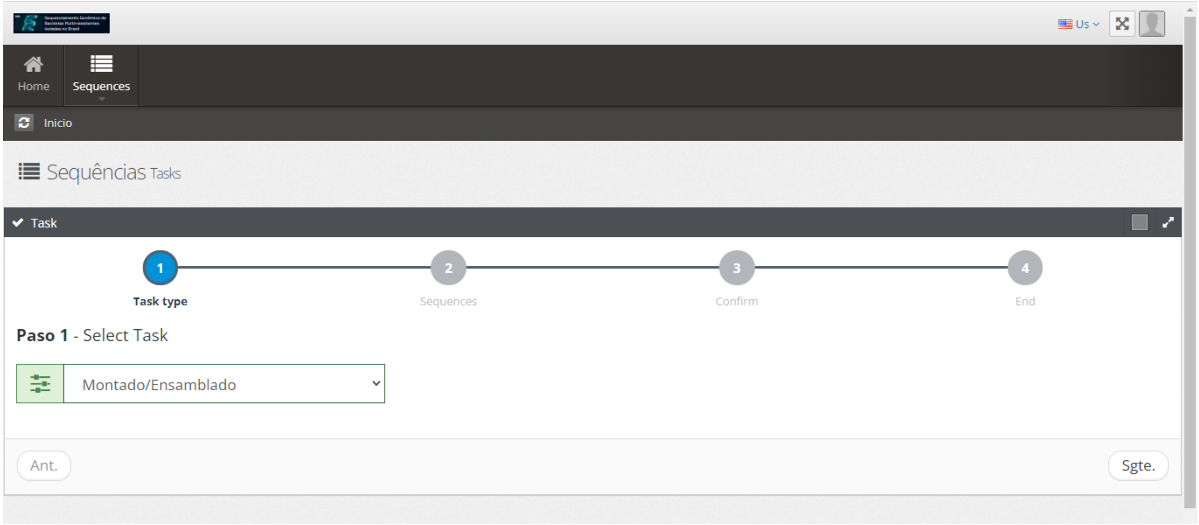

The user can also perform the two analyzes by selecting the all option.

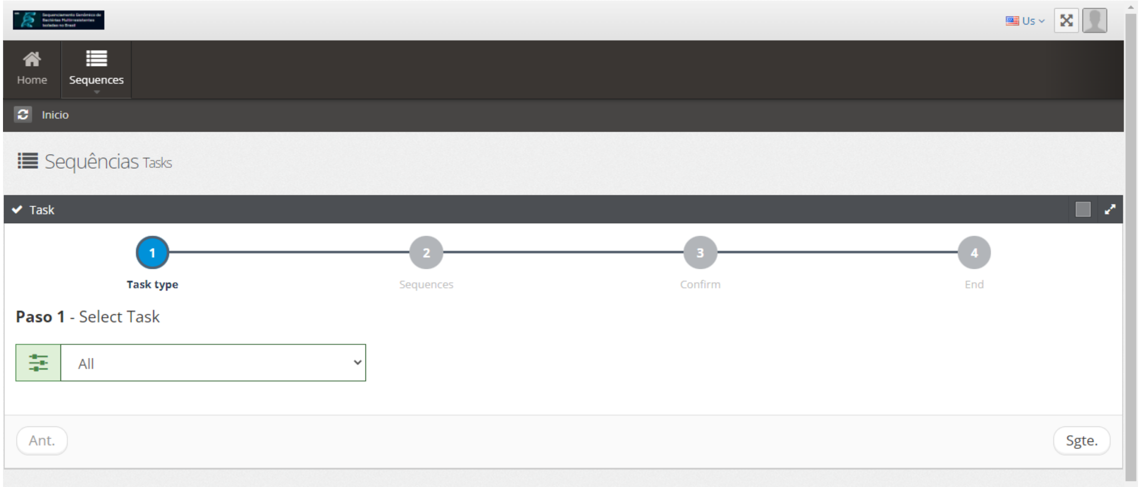

The next step, once the type of analysis to be performed has been selected, the user must select the sequence or sequences to be analyzed, for that they must click on the next icon 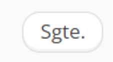

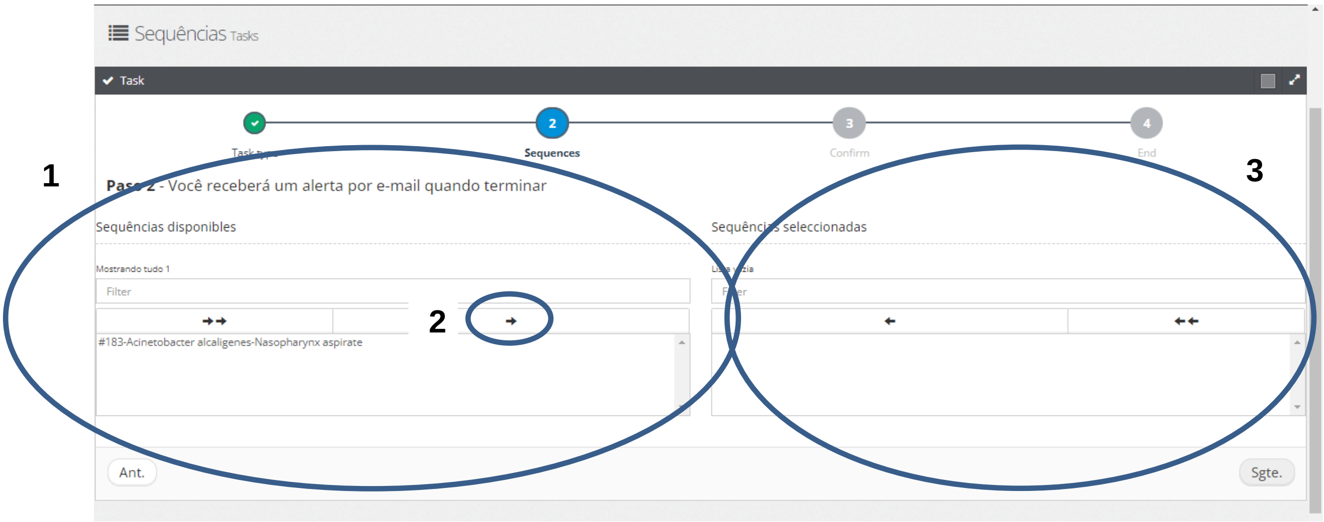

In this section, the user must select one or more sequences to be analyzed by clicking on the sequences, located in the available sequences box (1), once selected, they must click on the move selection icon (2).

When the sequences are moved and appear in the selected sequences box (3), click on the next icon. 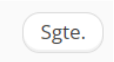

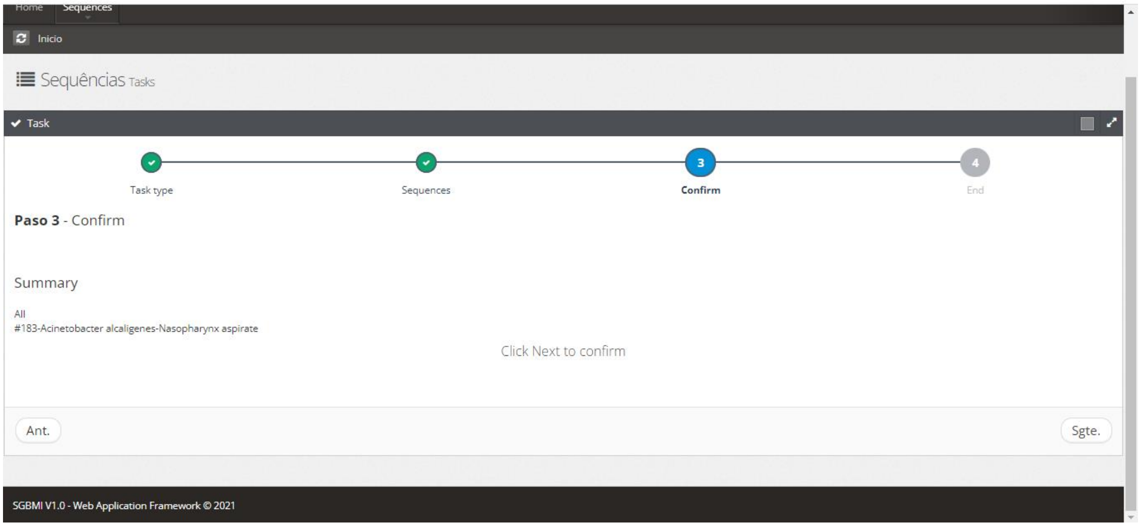

In this window you can confirm the selected sequences, if applicable, confirm by clicking on the next or previous button to modify the selection.

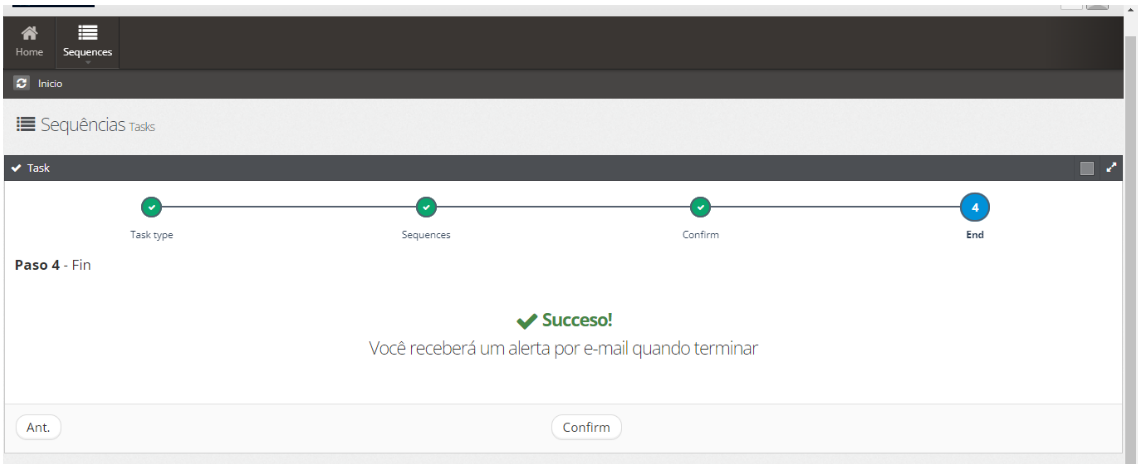

Once the task is confirmed. A message is displayed notifying the user that they will be notified via email when the selected analysis process starts and ends so that they can access the results section of the analysis performed. The process starts once analyzes prior to yours are finished.

c. Visualization of Results: Once the analysis is finished, the user is notified via email. He can see his results in the sequences section found in the Main Menu.

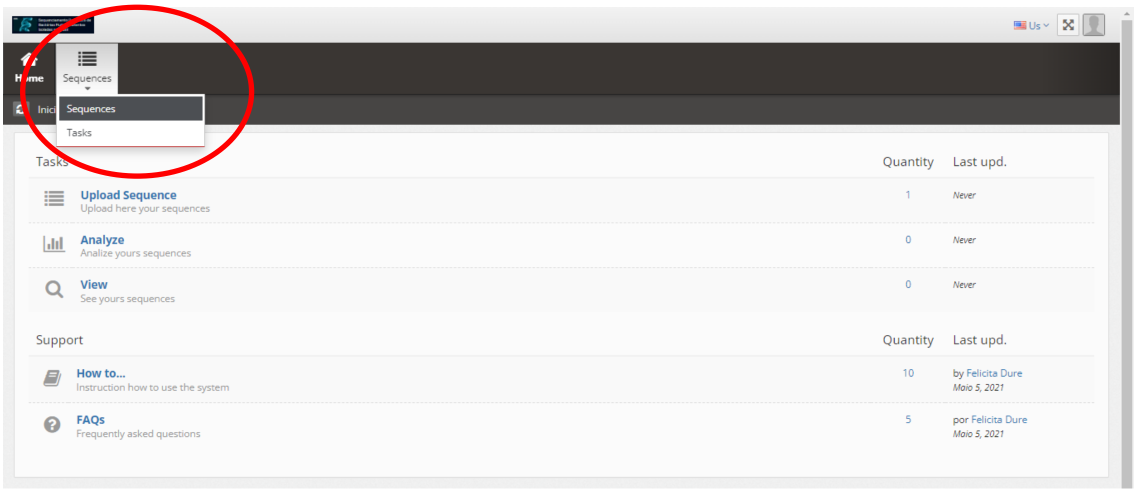

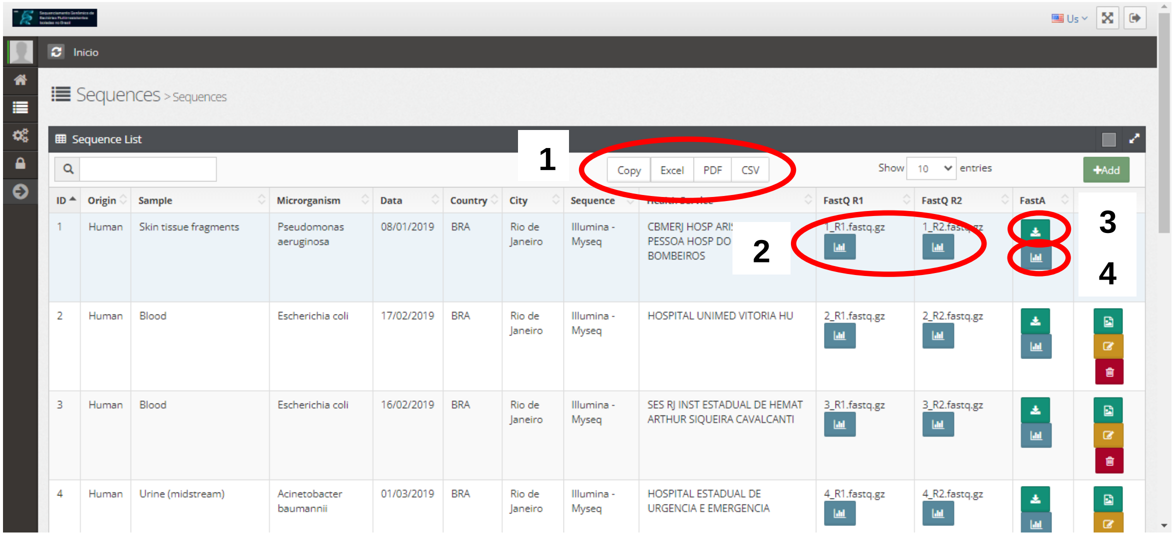

On this screen you can view the results of:

1. Streams Metadata
2. Quality Control Results (FASTQC)
3. Fasta File Generated
4. Individual result of each sequence of the processes carried out in Bioinformatic Analysis.

1- Metadata of the sequences: in this section you can download the data in four formats: Copy, in Excel, PDF and csv;

a. Copy and Excel: The data is downloaded in an Excel spreadsheet as follows

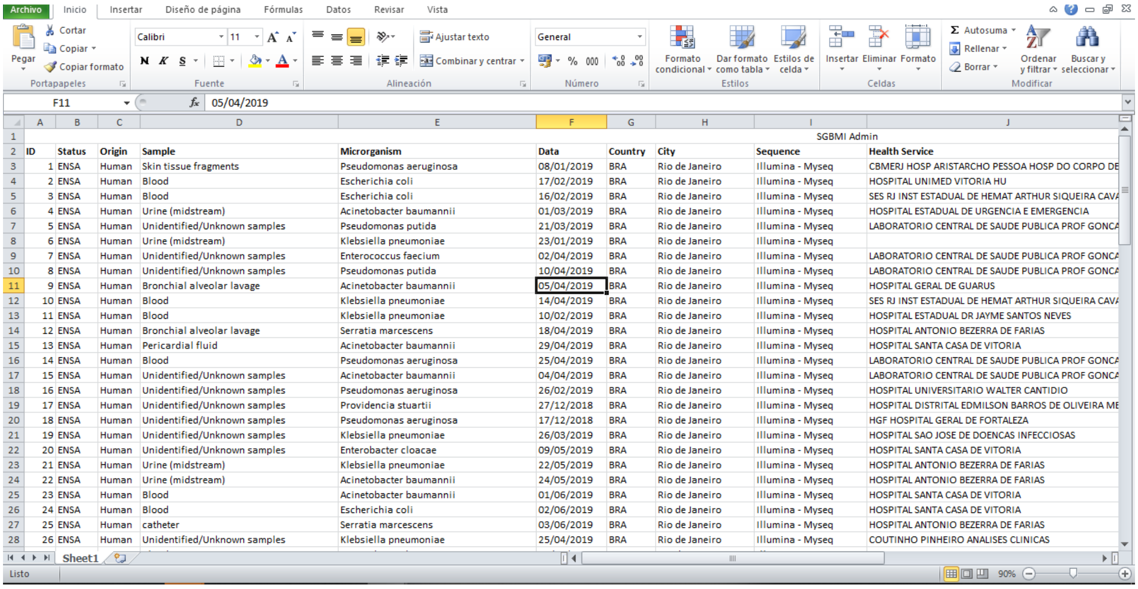

	A	B	C	D	E	F	G	H	I	J
1									SGBMI Admin	
2	ID	Status	Origin	Sample	Microrganism	Data	Country	City	Sequence	Health Service
3	1	ENSA	Human	Skin tissue fragments	Pseudomonas aeruginosa	08/01/2019	BRA	Rio de Janeiro	Illumina - Myseq	CBMERJ HOSP ARISTARCHO PESSOA HOSP DO CORPO DE
4	2	ENSA	Human	Blood	Escherichia coli	17/02/2019	BRA	Rio de Janeiro	Illumina - Myseq	HOSPITAL UNIMED VITORIA HU
5	3	ENSA	Human	Blood	Escherichia coli	16/02/2019	BRA	Rio de Janeiro	Illumina - Myseq	SES RJ INST ESTADUAL DE HEMAT ARTHUR SIQUEIRA CAVA
6	4	ENSA	Human	Urine (midstream)	Acinetobacter baumannii	01/03/2019	BRA	Rio de Janeiro	Illumina - Myseq	HOSPITAL ESTADUAL DE URGENCIA E EMERGENCIA
7	5	ENSA	Human	Unidentified/Unknown samples	Pseudomonas putida	21/03/2019	BRA	Rio de Janeiro	Illumina - Myseq	LABORATORIO CENTRAL DE SAUDE PUBLICA PROF GONCA
8	6	ENSA	Human	Urine (midstream)	Klebsiella pneumoniae	23/01/2019	BRA	Rio de Janeiro	Illumina - Myseq	
9	7	ENSA	Human	Unidentified/Unknown samples	Enterococcus faecium	02/04/2019	BRA	Rio de Janeiro	Illumina - Myseq	LABORATORIO CENTRAL DE SAUDE PUBLICA PROF GONCA
10	8	ENSA	Human	Unidentified/Unknown samples	Pseudomonas putida	10/04/2019	BRA	Rio de Janeiro	Illumina - Myseq	LABORATORIO CENTRAL DE SAUDE PUBLICA PROF GONCA
11	9	ENSA	Human	Bronchial alveolar lavage	Acinetobacter baumannii	05/04/2019	BRA	Rio de Janeiro	Illumina - Myseq	HOSPITAL GERAL DE GUARUS
12	10	ENSA	Human	Blood	Klebsiella pneumoniae	14/04/2019	BRA	Rio de Janeiro	Illumina - Myseq	SES RJ INST ESTADUAL DE HEMAT ARTHUR SIQUEIRA CAVA
13	11	ENSA	Human	Blood	Klebsiella pneumoniae	10/02/2019	BRA	Rio de Janeiro	Illumina - Myseq	HOSPITAL ESTADUAL DR JAYME SANTOS NEVES
14	12	ENSA	Human	Bronchial alveolar lavage	Serratia marcescens	18/04/2019	BRA	Rio de Janeiro	Illumina - Myseq	HOSPITAL ANTONIO BEZERRA DE FARIAS
15	13	ENSA	Human	Pericardial fluid	Acinetobacter baumannii	29/04/2019	BRA	Rio de Janeiro	Illumina - Myseq	HOSPITAL SANTA CASA DE VITORIA
16	14	ENSA	Human	Blood	Pseudomonas aeruginosa	25/04/2019	BRA	Rio de Janeiro	Illumina - Myseq	LABORATORIO CENTRAL DE SAUDE PUBLICA PROF GONCA
17	15	ENSA	Human	Unidentified/Unknown samples	Acinetobacter baumannii	04/04/2019	BRA	Rio de Janeiro	Illumina - Myseq	LABORATORIO CENTRAL DE SAUDE PUBLICA PROF GONCA
18	16	ENSA	Human	Unidentified/Unknown samples	Pseudomonas aeruginosa	26/02/2019	BRA	Rio de Janeiro	Illumina - Myseq	HOSPITAL UNIVERSITARIO WALTER CANTIDIO
19	17	ENSA	Human	Unidentified/Unknown samples	Providencia stuartii	27/12/2018	BRA	Rio de Janeiro	Illumina - Myseq	HOSPITAL DISTRITAL EDMILSON BARROS DE OLIVEIRA ME
20	18	ENSA	Human	Unidentified/Unknown samples	Pseudomonas aeruginosa	17/12/2018	BRA	Rio de Janeiro	Illumina - Myseq	HGF HOSPITAL GERAL DE FORTALEZA
21	19	ENSA	Human	Unidentified/Unknown samples	Klebsiella pneumoniae	26/03/2019	BRA	Rio de Janeiro	Illumina - Myseq	HOSPITAL SAO JOSE DE DOENCAS INFECCIOSAS
22	20	ENSA	Human	Unidentified/Unknown samples	Enterobacter cloacae	09/05/2019	BRA	Rio de Janeiro	Illumina - Myseq	HOSPITAL SANTA CASA DE VITORIA
23	21	ENSA	Human	Urine (midstream)	Klebsiella pneumoniae	22/05/2019	BRA	Rio de Janeiro	Illumina - Myseq	HOSPITAL ANTONIO BEZERRA DE FARIAS
24	22	ENSA	Human	Urine (midstream)	Acinetobacter baumannii	24/05/2019	BRA	Rio de Janeiro	Illumina - Myseq	HOSPITAL ANTONIO BEZERRA DE FARIAS
25	23	ENSA	Human	Blood	Acinetobacter baumannii	01/06/2019	BRA	Rio de Janeiro	Illumina - Myseq	HOSPITAL SANTA CASA DE VITORIA
26	24	ENSA	Human	Blood	Escherichia coli	02/06/2019	BRA	Rio de Janeiro	Illumina - Myseq	HOSPITAL SANTA CASA DE VITORIA
27	25	ENSA	Human	catheter	Serratia marcescens	03/06/2019	BRA	Rio de Janeiro	Illumina - Myseq	HOSPITAL ANTONIO BEZERRA DE FARIAS
28	26	ENSA	Human	Unidentified/Unknown samples	Klebsiella pneumoniae	25/04/2019	BRA	Rio de Janeiro	Illumina - Myseq	COUTINHO PINHEIRO ANALISES CLINICAS

b. PDF: in pdf format as follows

SGBMI Admin

ID	Estado	Origin	Sample	Hospedeiro	Microrganism	Data	Country	City	Sequence	H
1	ENSA	Human	Skin tissue fragments	.	Pseudomonas aeruginosa	08/01/2019	BRA	Rio de Janeiro	Illumina - Myseq	CE AF PE DC BC
2	ENSA	Human	Blood	.	Escherichia coli	17/02/2019	BRA	Rio de Janeiro	Illumina - Myseq	HC UN HU
3	ENSA	Human	Blood	.	Escherichia coli	16/02/2019	BRA	Rio de Janeiro	Illumina - Myseq	SE ES HE SI CA
4	ENSA	Human	Urine (midstream)	.	Acinetobacter baumannii	01/03/2019	BRA	Rio de Janeiro	Illumina - Myseq	HC ES UF EN
5	ENSA	Human	Unidentified/ Unknown samples	.	Pseudomonas putida	21/03/2019	BRA	Rio de Janeiro	Illumina - Myseq	LA CE SA PF M
6	ENSA	Human	Urine (midstream)	.	Klebsiella pneumoniae	23/01/2019	BRA	Rio de Janeiro	Illumina - Myseq	
7	ENSA	Human	Unidentified/ Unknown samples	.	Enterococcus faecium	02/04/2019	BRA	Rio de Janeiro	Illumina - Myseq	LA CE SA PF M

c. CSV (comma separated file) downloaded as follows

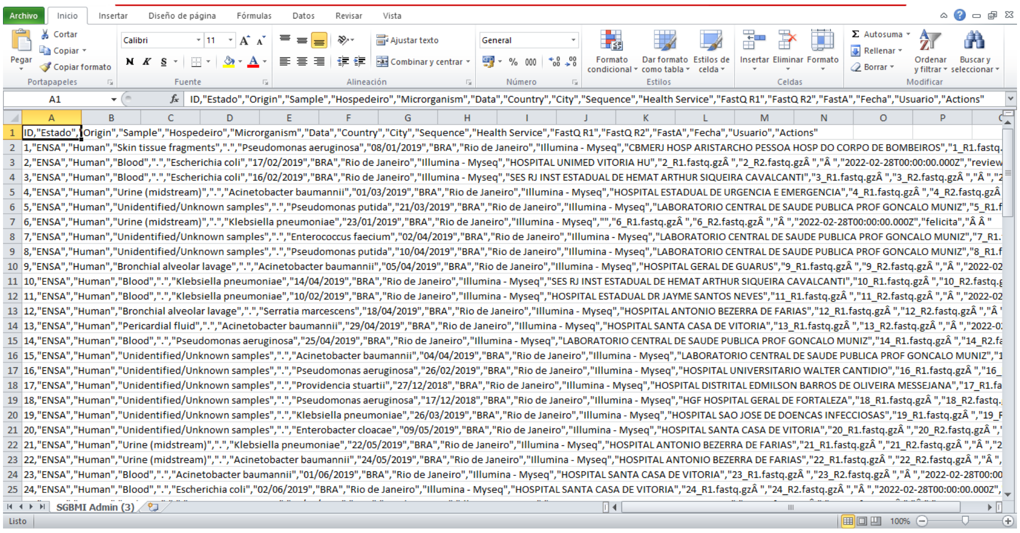

2. **Quality Control Results (FASTQC):** In the line of each sequence an icon appears that links to an .html file that is the result report issued by the FASTQC program where several quality control points are analyzed.

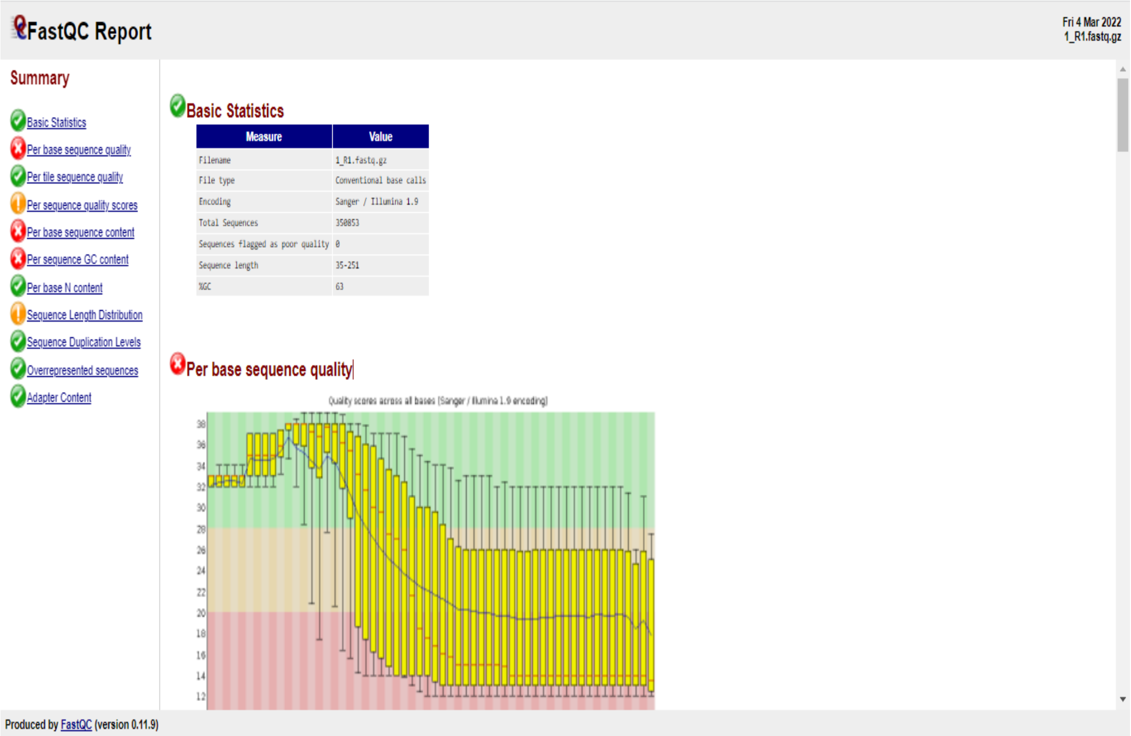

3. Fasta File Generated

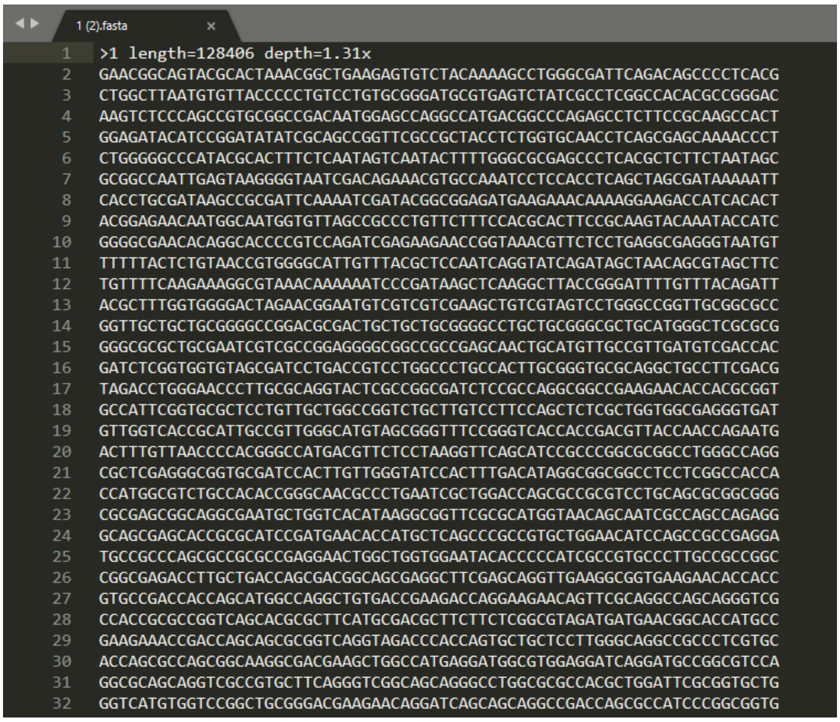

1 (2).fasta

```
>1 length=128406 depth=1.31x
GAACGGCAGTACGCACTAAACGGCTGAAGAGTGTCTACAAAAGCCTGGGCGATTCAGACAGCCCCTCACG
CTGGCTTAATGTGTTACCCCCTGTCCTGTGCGGGATGCGTGAGTCTATCGCCTCGGCCACACGCCGGGAC
AAGTCTCCCAGCCGTGCGGCCGACAATGGAGCCAGGCCATGACGGCCCAGAGCCTCTTCCGCAAGCCACT
GGAGATACATCCGGATATATCGCAGCCGGTTCGCCGCTACCTCTGGTGCAACCTCAGCGAGCAAAACCCT
CTGGGGGCCCATACGCACTTTCTCAATAGTCAATACTTTTGGGCGCGAGCCCTCACGCTCTTCTAATAGC
GCGGCCAATTGAGTAAGGGGTAATCGACAGAAACGTGCCAAATCCTCCACCTCAGCTAGCGATAAAAATT
CACCTGCGATAAGCCGCGATTCAAAATCGATACGGCGGAGATGAAGAAACAAAAGGAAGACCATCACACT
ACGGAGAACAATGGCAATGGTGTTAGCCGCCCTGTTCTTTCCACGCACTTCCGCAAGTACAAATACCATC
GGGGCGAACACAGGCACCCCGTCCAGATCGAGAAGAACCGGTAAACGTTCTCCTGAGGCGAGGGTAATGT
TTTTTACTCTGTAACCGTGGGGCATTGTTTACGCTCCAATCAGGTATCAGATAGCTAACAGCGTAGCTTC
TGTTTTCAAGAAAGGCGTAAACAAAAAATCCCGATAAGCTCAAGGCTTACCGGGATTTTGTTTACAGATT
ACGCTTTGGTGGGGACTAGAACGGAATGTCGTCGTCGAAGCTGTCGTAGTCCTGGGCCGGTTGCGGCGCC
GGTTGCTGCTGCGGGGCCGGACGCGACTGCTGCTGCGGGGCCTGCTGCGGGCGCTGCATGGGCTCGCGCG
GGGCGCGCTGCGAATCGTCGCCGGAGGGGCGGCCGCCGAGCAACTGCATGTTGCCGTTGATGTCGACCAC
GATCTCGGTGGTGTAGCGATCCTGACCGTCCTGGCCCTGCCACTTGCGGGTGCGCAGGCTGCCTTCGACG
TAGACCTGGGAACCCTTGCGCAGGTACTCGCCGGCGATCTCCGCCAGGCGGCCGAAGAACACCACGCGGT
GCCATTCGGTGCGCTCCTGTTGCTGGCCGGTCTGCTTGTCCTTCCAGCTCTCGCTGGTGGCGAGGGTGAT
GTTGGTCACCGCATTGCCGTTGGGCATGTAGCGGGTTTCCGGGTCACCACCGACGTTACCAACCAGAATG
ACTTTGTTAACCCCACGGGCCATGACGTTCTCCTAAGGTTCAGCATCCGCCCGGCGCGGCCTGGGCCAGG
CGCTCGAGGGCGGTGCGATCCACTTGTTGGGTATCCACTTTGACATAGGCGGCGGCCTCCTCGGCCACCA
CCATGGCGTCTGCCACACCGGGCAACGCCCTGAATCGCTGGACCAGCGCCGCGTCCTGCAGCGCGGCGGG
CGCGAGCGGCAGGCGAATGCTGGTCACATAAGGCGGTTCGCGCATGGTAACAGCAATCGCCAGCCAGAGG
GCAGCGAGCACCGCGCATCCGATGAACACCATGCTCAGCCCGCCGTGCTGGAACATCCAGCCGCCGAGGA
TGCCGCCCAGCGCCGCGCCGAGGAACTGGCTGGTGGAATACACCCCCATCGCCGTGCCCTTGCCGCCGGC
CGGCGAGACCTTGCTGACCAGCGACGGCAGCGAGGCTTCGAGCAGGTTGAAGGCGGTGAAGAACACCACC
GTGCCGACCACCAGCATGGCCAGGCTGTGACCGAAGACCAGGAAGAACAGTTCGCAGGCCAGCAGGGTCG
CCACCGCGCCGGTCAGCACGCGCTTCATGCGACGCTTCTTCTCGGCGTAGATGATGAACGGCACCATGCC
GAAGAAACCGACCAGCAGCGCGGTCAGGTAGACCCACCAGTGCTGCTCCTTGGGCAGGCCGCCCTCGTGC
ACCAGCGCCAGCGGCAAGGCGACGAAGCTGGCCATGAGGATGGCGTGGAGGATCAGGATGCCGGCGTCCA
GGCGCAGCAGGTCGCCGTGCTTCAGGGTCGGCAGCAGGGCCTGGCGCGCCACGCTGGATTCGCGGTGCTG
GGTCATGTGGTCCGGCTGCGGGACGAAGAACAGGATCAGCAGCAGGCCGACCAGCGCCATCCCGGCGGTG
```

4. Individual result of each sequence of the processes carried out in Bioinformatic Analysis. These results can be viewed on the screen as well as downloaded in Excel by clicking the Download in Excel button.

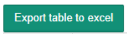

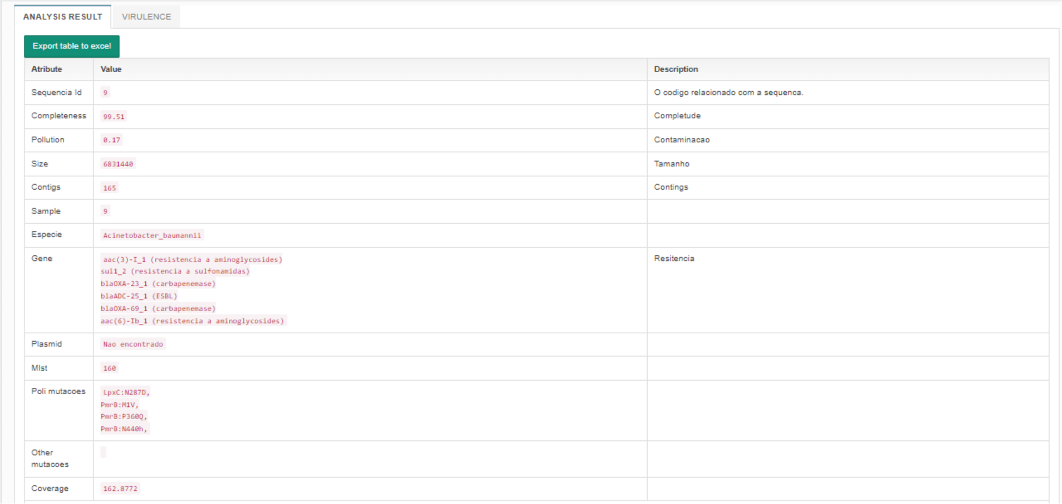

ANALYSIS RESULT | VIRULENCE

Export table to excel

Atribute	Value	Description
Sequencia Id	9	O codigo relacionado com a sequenca.
Completeness	99.51	Completude
Pollution	0.17	Contaminacao
Size	6831440	Tamanho
Contigs	165	Contings
Sample	9	
Especie	Acinetobacter_baumannii	
Gene	aac(3)-I_1 (resistencia a aminoglycosides)<br>sul1_2 (resistencia a sulfonamidas)<br>blaOXA-23_1 (carbapenemase)<br>blaADC-25_1 (ESBL)<br>blaOXA-69_1 (carbapenemase)<br>aac(6)-Ib_1 (resistencia a aminoglycosides)	Resitencia
Plasmid	Nao encontrado	
Mlst	160	
Poli mutacoes	LpxC:N287D,<br>PmrB:M1V,<br>PmrB:P360Q,<br>PmrB:N440h,	
Other mutacoes		
Coverage	162.8772	

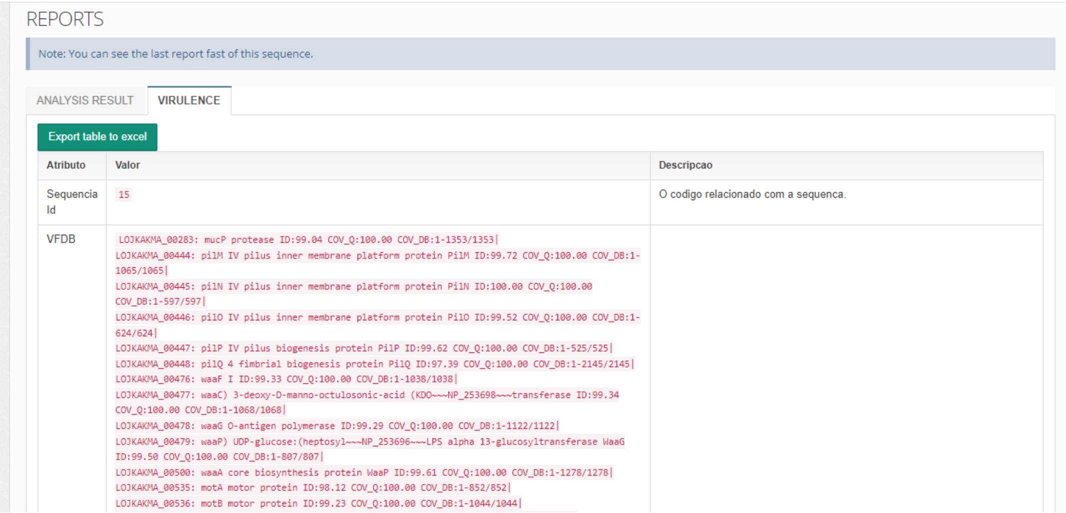

5.3 Views:

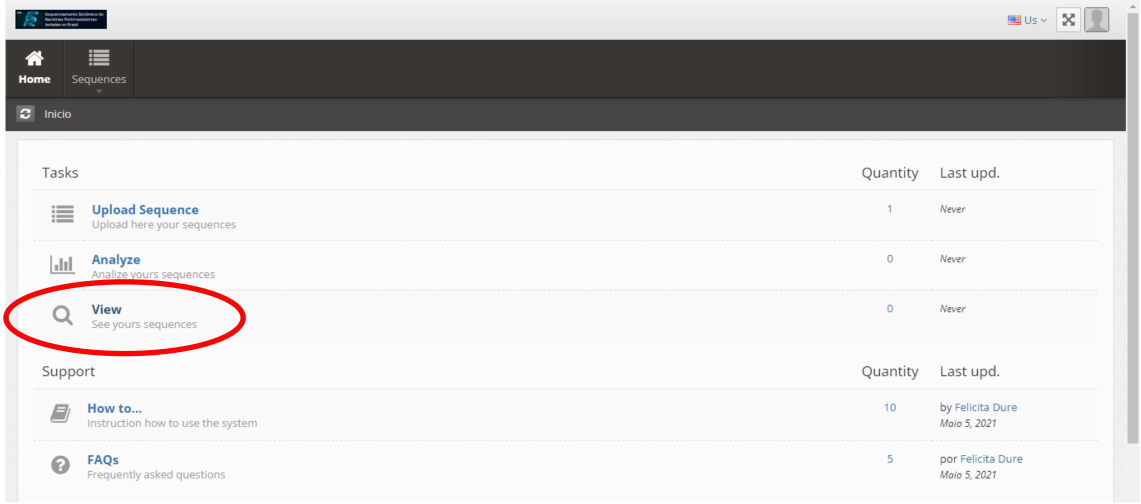

In this section the user can access the sequenced data that is in the database as a Reference, the visualization is done with the Microreact program, which allows visualizing and exploring any combination of grouping data (trees), geographic (map) and temporal (timeline). The available variables are displayed in a table.

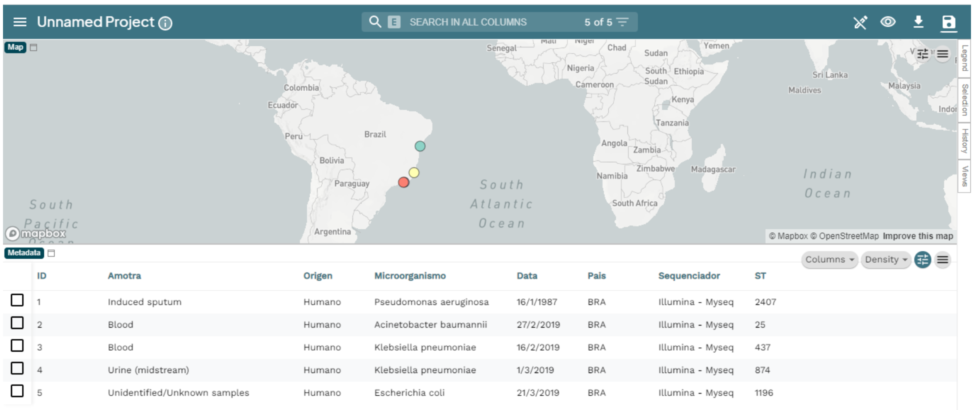

6. **Support:** This section is designed to guide the user on how to use the system. Two-part account: How: in which the user can access the CABGen System User Manual and the Frequently Asked Questions, which consists of a forum in which users can interact with the System administrators.

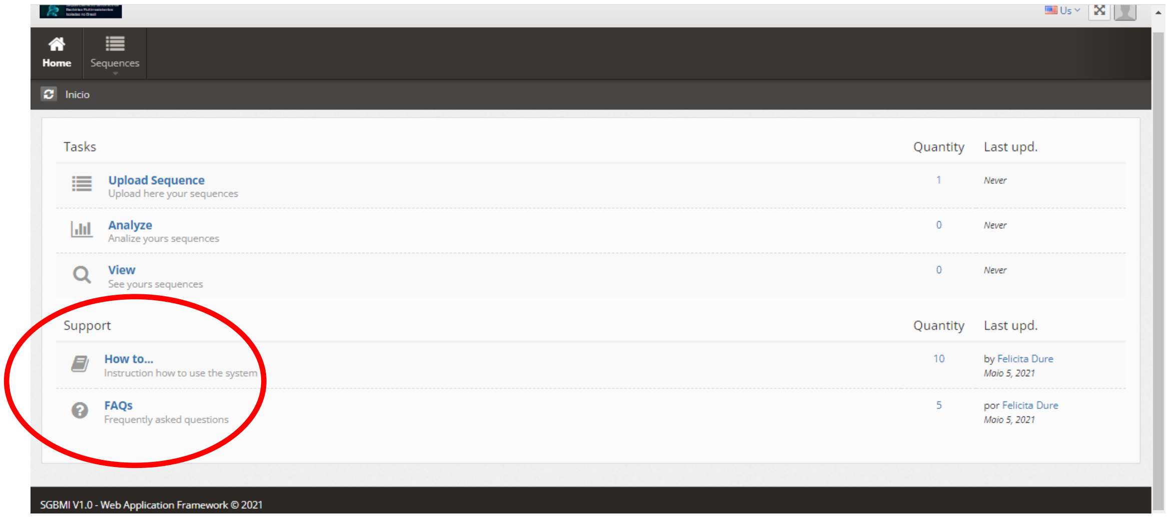

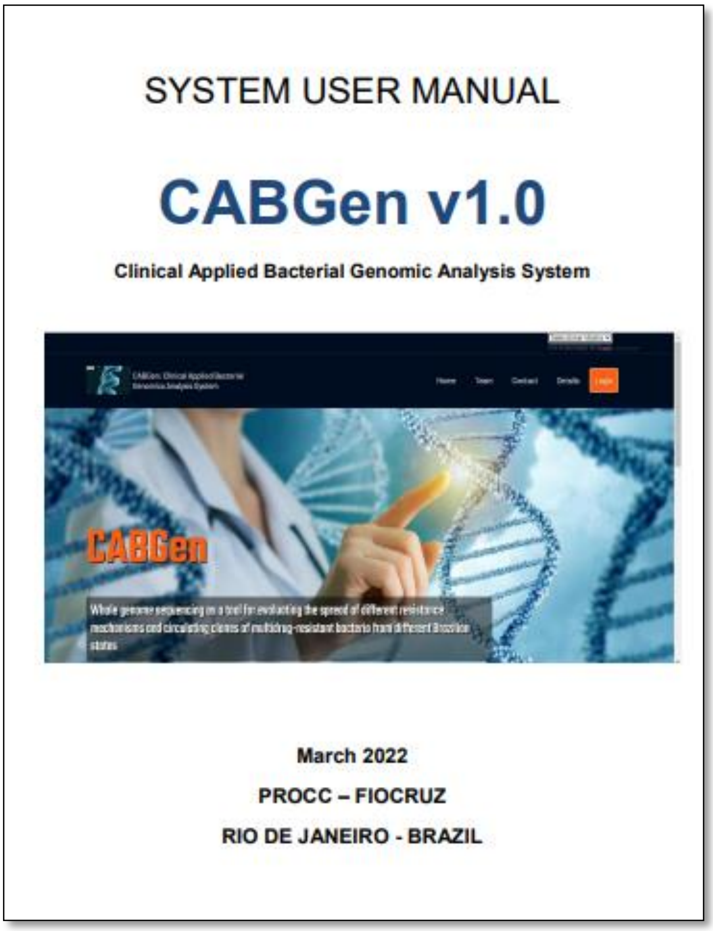

Supplement: Supplementary file 2 [file Data_Sheet_1.PDF]
